# Lattice Strain and Piezoelectric Field Modulated Piezo‐Photocatalytic Nitrate‐to‐Ammonia Conversion in Chemically Bonded S‐Scheme Heterojunction

**DOI:** 10.1002/advs.202518794

**Published:** 2025-11-19

**Authors:** Shuo Liu, Jing Li, Zhiwei Liu, Jiaxuan Song, Huijun Lv, Dongdong Xiao, Qizhao Wang, Yongzheng Zhang, Qikun Xue

**Affiliations:** ^1^ School of Physics and Physical Engineering Qufu Normal University Qufu 273165 China; ^2^ College of Chemistry and Chemical Engineering Northwest Normal University Lanzhou 730070 China; ^3^ Technical Institute of Physics and Chemistry Chinese Academy of Sciences Beijing 100190 China; ^4^ Quantum Science Center of Guangdong‐Hong Kong‐Macao Greater Bay Area Shenzhen 518102 China; ^5^ Institute of Physics Chinese Academy of Sciences Beijing 100190 China

**Keywords:** chemically bonded, lattice strain, nitrate‐to‐ammonia, piezo‐photocatalytic, S‐scheme heterojunction

## Abstract

Piezo‐photocatalysis provides an efficient approach to harness mechanical and solar energy simultaneously for chemical conversion. However, prior studies predominantly focus on piezoelectric fields, while ignoring the influence of lattice strain induced by mechanical stimulations in the piezo‐photocatalytic process. Herein, a ZnO@ZnSe S‐scheme heterojunction is constructed by in situ growth of an ultrathin ZnSe shell on piezoelectric ZnO nanorods for piezo‐photocatalytic nitrate‐to‐ammonia. Such a piezo‐photoelectric heterojunction is designed to exploit the lattice strain and piezoelectric effect in parallel. Under ultrasound irradiation, the coherent lattice strain induced by O─Zn─Se interfacial chemical bonds regulates the active‐site Zn and Se atoms in the ZnSe shell, accelerating the dissociation of H_2_O molecules at Zn atoms and optimizing the thermodynamic process of nitrate‐to‐ammonia at Se atoms. Meanwhile, the piezoelectric field in ZnO modulates the band structure of ZnO@ZnSe heterojunction, enabling sustained charge carrier separation. Benefiting from the synergistic effects, the ZnO@ZnSe catalyst achieved an ammonia yield of 2.88 mmol g^−1^ h^−1^ and a high selectivity of 92.86% in the piezo‐photocatalytic nitrate‐to‐ammonia process. This work reveals, for the first time, the synergy between the piezoelectric field and lattice strain in piezo‐photocatalysis, paving the way for designing advanced catalytic systems based on a novel principle.

## Graphical abstract

Under ultrasonic excitation, 5% compressive strain optimizes the ZnO@ZnSe heterojunction. The strain enhances H_2_O dissociation into active ^*^H on Zn sites and NO_3_^−^ adsorption on Se sites, facilitating their hydrogenation into NH_4_^+^ with optimal kinetics and thermodynamics.

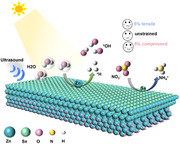

## 1 Introduction

Nitrate (NO_3_^−^) is a common pollutant in agriculture, industry, and daily life.^[^ ^1^ ^]^ Compared with the N≡N bond (941 kJ mol^−1^) in nitrogen (N_2_), the N═O bond (204 kJ mol^−1^) in NO_3_^−^ possesses significantly lower dissociation energy, making NO_3_^−^ a more reactive and accessible nitrogen source.^[^ ^2^ ^]^ As such, NO_3_^−^ has emerged as a promising alternative nitrogen source to N_2_ for ammonia (NH_3_) synthesis, achieving the dual benefits of environmental remediation and value‐added chemical production.^[^ ^3^, ^4^ ^]^ Extensive research has highlighted the photocatalytic reduction of NO_3_^−^ to NH_3_ as a highly efficient, low‐energy‐consuming, and environmentally friendly approach under mild reaction conditions.^[^ ^5^ ^]^

However, photocatalytic NO_3_^−^ reduction to NH_3_ is hindered by the challenges beyond carrier separation efficiency, including sluggish reaction kinetics and the formation of undesirable byproducts, due to a complex eight‐electron/nine‐proton transfer process and multiple intermediate adsorption/desorption steps.^[^ ^6^ ^]^ Previous studies have demonstrated that modulating the electronic structure and density of states of catalytic active sites can effectively optimize intermediate adsorption and activation energies of key intermediates, thereby facilitating ^*^H incorporation and selectively steering the reaction pathway toward NH_3_ while suppressing competing side reactions.^[^ ^7^, ^8^ ^]^ Among various strategies for regulating catalytic active sites, the lattice strain effect has emerged as a particularly effective approach. Lattice strain, tensile or compressive strain within the catalyst's crystal lattice, is usually induced by epitaxial growth‐induced mismatch, interfacial lattice constant disparities, or externally applied mechanical stress,^[^ ^9^, ^10^ ^]^ which can significantly alter the electronic structure and density of states at active sites, thereby modulating the adsorption/desorption behavior of reactants and intermediates to favor optimized reaction pathways and improved catalytic performance.^[^ ^11^, ^12^ ^]^

Coincidentally, the lattice strain is consequently induced by mechanical deformation in a piezoelectric system during ultrasonic cavitation or stirring.^[^ ^13^ ^]^ However, in previous studies on piezoelectric catalysis, the influence of the lattice strain induced by mechanical stimulation on the entire system had not been addressed. Furthermore, owing to the non‐centrosymmetric structure of piezoelectric materials, external mechanical stimulation can induce a piezoelectric field through the displacement of positive and negative charge centers.^[^ ^14^, ^15^ ^]^ This field dynamically modulates electron transfer pathways, thereby enhancing charge carrier separation both within the bulk and at the surface of the semiconductor, which can induce interfacial band bending, effectively tuning the built‐in electric field of the heterojunction to promote more efficient photogenerated carrier separation.^[^ ^16^, ^17^ ^]^ Thus, highly efficient carrier separation caused by its unique carrier transfer mechanism, as well as the synergistic effect of lattice strain and piezoelectric field, can be achieved in the piezoelectric S‐scheme heterojunctions.^[^ ^18^, ^19^ ^]^

Therefore, a key challenge in piezocatalysis is how the lattice strain and piezoelectric field generated under mechanical stimulation can be harnessed in tandem to regulate both active sites and the electronic band structure. Herein, a piezo‐photoelectric ZnO@ZnSe S‐scheme heterojunction by in situ growth of an ≈5‐nanometer‐thick ultrathin ZnSe shell on 1D ZnO nanorods was successfully fabricated in this work. At the heterojunction interface, ZnO and ZnSe are connected via the interfacial O─Zn─Se chemical bond formed through bridging Zn atoms, enabling ZnSe to exhibit synchronized mechanical responses under applied mechanical stress. This rational design allows the ZnO@ZnSe catalyst to exhibit dual functionalities under mechanical stimulation. The piezoelectric ZnO material generates a piezoelectric field that induces band bending within the S‐scheme heterojunction, thereby enhancing carrier separation and migration of photogenerated charge carriers. Simultaneously, the interfacial O─Zn─Se chemical bonding induces synchronized lattice strain within the ultrathin ZnSe shell, which accelerates the speed of H_2_O dissociation on Zn atoms to promote ^*^H generation, increases the density of states for the active‐site Se atoms near the Fermi level, enhances the Se‐N orbital interaction between the Se atom and the critical intermediate ^*^NOH, and reduces the energy barrier of the rate‐determining step (^*^NO to ^*^NOH), thus improving the reaction thermodynamics. These two synergistic effects induced by ultrasound stimulation enable the rationally designed ZnO@ZnSe S‐scheme heterojunction catalyst to achieve a NH_4_^+^ production rate of 2.88 mmol g^−1^ h^−1^ and a selectivity of 92.86% in a neutral and mild sacrificial agent sodium formate (HCOONa) system. This work not only elucidates the regulatory role of the piezoelectric field in modulating the heterojunction band structure but, more importantly, reveals the critical influence of mechanically induced lattice strain on active site performance. These insights offer significant mechanistic understanding for the design of efficient piezo‐photoelectric catalysts for other catalytic reactions.

## 2 Results and Discussion

### 2.1 Synthesis and Structural Characterization of Catalysts

**Figure 1:**
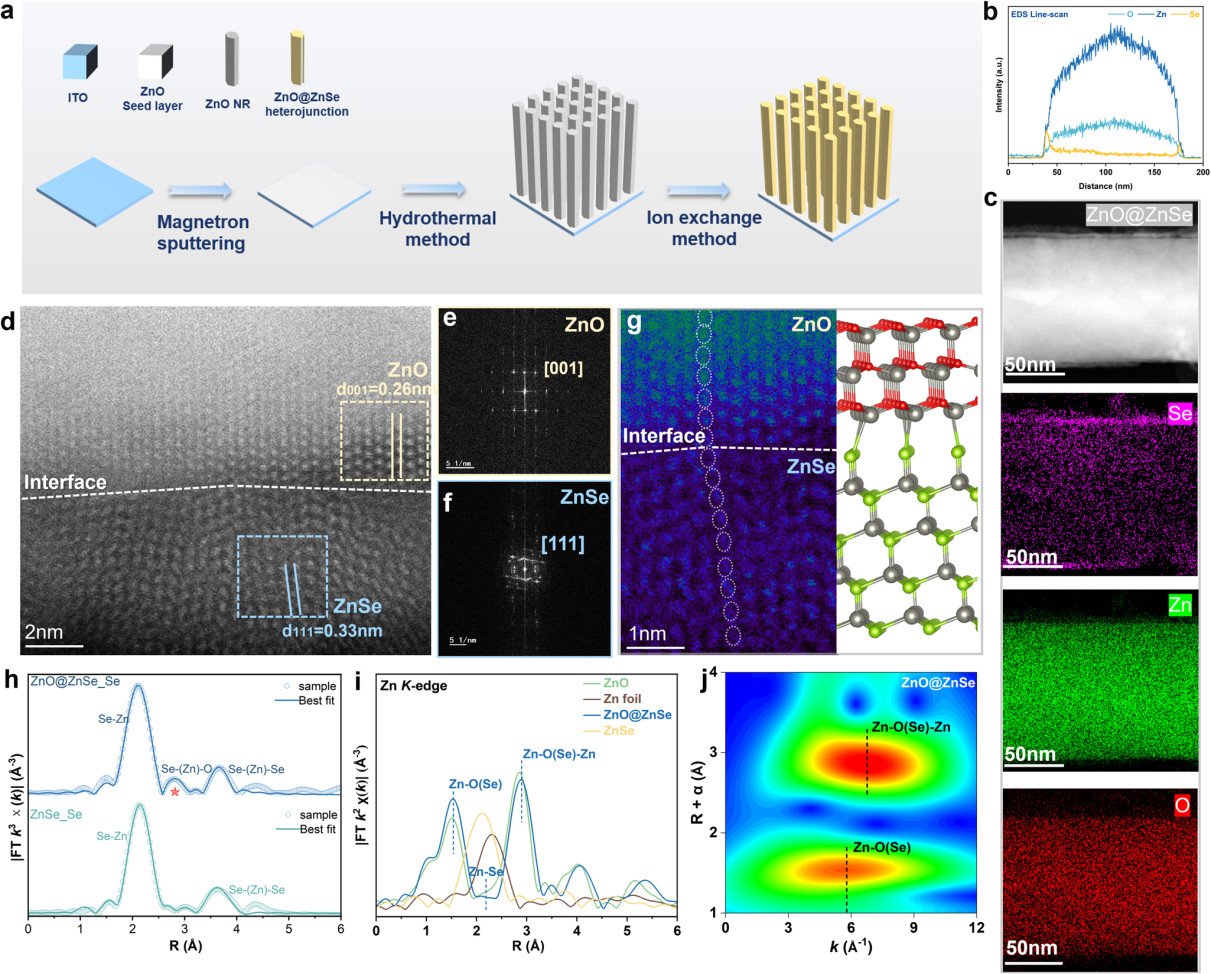
a) ZnO@ZnSe synthesis pathway. b) EDS line‐scan result. c) HAADF‐STEM image at the 50 nm scale, corresponding element (Zn, O, and Se) mappings of ZnO@ZnSe. d) HAADF‐STEM image of the ZnO@ZnSe heterointerface at the 2 nm scale. e,f) Fast Fourier transform patterns of ZnO and ZnSe. g) HAADF‐STEM image of the heterointerface at the 1 nm scale and ZnO@ZnSe heterojunction model (Note: Gray represents Zn atoms, green represents Se atoms, and red represents O atoms). h) The normalized Se K‐edge FT‐EXAFS curves of ZnO@ZnSe and ZnSe. i) The normalized Zn K‐edge FT‐EXAFS curves of Zn foil, ZnO, ZnO@ZnSe, and ZnSe. j) The WT‐EXAFS of Zn K‐edge for ZnO@ZnSe.

The whole schematic diagram of the synthesis process for ZnO@ZnSe heterojunction is displayed in **Figure** **1**a. Uniform and well‐oriented ZnO nanorods (Figures S1 and S2, Supporting Information) were synthesized by a magnetron sputtering‐assisted hydrothermal method.^[^ ^20^ ^]^ Based on the solubility product constants (*K*_sp_) difference between ZnO and ZnSe, an ≈5‐nm‐thick ZnSe shell was in situ grown on the ZnO surface via ion exchange in a Se^2−^‐rich environment, forming ZnO@ZnSe heterojunction.^[^ ^21^ ^]^ Scanning electron microscopy (SEM) confirmed that the morphology of the ZnO@ZnSe heterojunction remained unchanged after Se^2^^−^ ion exchange compared to the initial ZnO structure. Energy dispersive spectroscopy (EDS) further revealed the homogeneous distribution of Zn, O, and Se elements across the sample (Figure S3, Supporting Information).

To characterize the material structure of the ZnO@ZnSe heterojunction and the atomic arrangement at the interface, high‐angle annular dark‐field scanning transmission electron microscopy (HAADF‐STEM) characterization was conducted. As shown in the EDS line scan and EDS (Figure 1b,c), Se is mainly concentrated in the shell of ZnO@ZnSe, indicating the successful growth of the ZnSe shell. In the high‐magnification HAADF‐STEM image (Figure 1d), distinct atomic arrangements were observed on either side of the heterointerface. Fast Fourier Transform (FFT) analysis of selected regions from both sides of the interface clearly demonstrated that the ZnO core crystallizes in a hexagonal wurtzite structure aligned along the [001] zone axis (Figure 1e), while the ZnSe shell possesses a cubic sphalerite structure with [111] orientation (Figure 1f).^[^ ^22^ ^]^ The HAADF‐STEM image of the heterointerface was magnified (Figure 1g), revealing a closely packed and continuous arrangement of Zn atoms at the interface.

To confirm the formation of the O─Zn─Se interfacial chemical bond, X‐ray absorption fine structure (XAFS) analysis was performed on Se and Zn elements. In the ZnO@ZnSe heterojunction, the Fourier‐transform EXAFS of the Se K‐edge reveals a new scattering signal at ≈2.7 Å in the second shell (Figure S4b, Supporting Information), which cannot be fitted solely using the Se‐Zn and Se‐Zn‐Se scattering paths of pure ZnSe. By introducing the Se‐O‐Se path (Figure 1h; Table S1, Supporting Information), the signal is well fitted, indicating backscattering contributions from O atoms around the bridging Zn atom, attributable to the formation of an O─Zn─Se bond. Moreover, the oscillation of Se in *k*‐space shifts toward lower *k*‐regions (Figure S4c, Supporting Information), and its wavelet‐transform EXAFS (WT‐EXAFS) further shows distinct signal sources in both high and low‐*k* regions for the second shell of Se in ZnO@ZnSe (Figure S5, Supporting Information), consistent with the Se K‐edge FT‐EXAFS results, indicating the presence of lighter O atoms around Se.

Analysis of the Zn FT‐EXAFS shows that the spectrum of ZnO@ZnSe closely matches that of pure ZnO (Figure 1i; Table S2, Supporting Information). However, the Zn‐O and Zn‐O‐Zn coordination bonds are slightly elongated, and the maximum oscillation peak of Zn in *k*‐space shifts toward higher *k* (Figure S6a,b, Supporting Information).^[^ ^23^ ^]^ These observations suggest the presence of Se, a stronger backscattering atom, around Zn. Combined with Zn K‐edge WT‐EXAFS results of ZnO and ZnO@ZnSe (Figure 1j; Figure S6c,d, Supporting Information), the intensity peaks of the first‐shell Zn‐O and second‐shell Zn‐O‐Zn bonds shift toward higher *k* regions.^[^ ^24^ ^]^ This shift, analyzed together with Zn *k*‐space EXAFS and K‐edge FT‐EXAFS, is attributed to the enhanced scattering effect of the heavier Se atom in the Zn─O(Se) and Zn─O(Se)‐Zn coordination environments at higher *k*.^[^ ^25^ ^]^ In summary, the changes in the coordination environments of Se and Zn revealed by XAFS analysis confirm the formation of O─Zn─Se chemical bonds at the interface.

Accordingly, a piezoelectric ZnO@ZnSe heterojunction with interfacial chemical bonds was successfully constructed. The interfacial O─Zn─Se chemical bonds act as “adhesives”, ensuring that ZnO and ZnSe maintain synchronous lattice strain under mechanical stimulation.

### 2.2 Charge Transfer Mechanisms and Energy Band Structures in Catalysts

**Figure 2:**
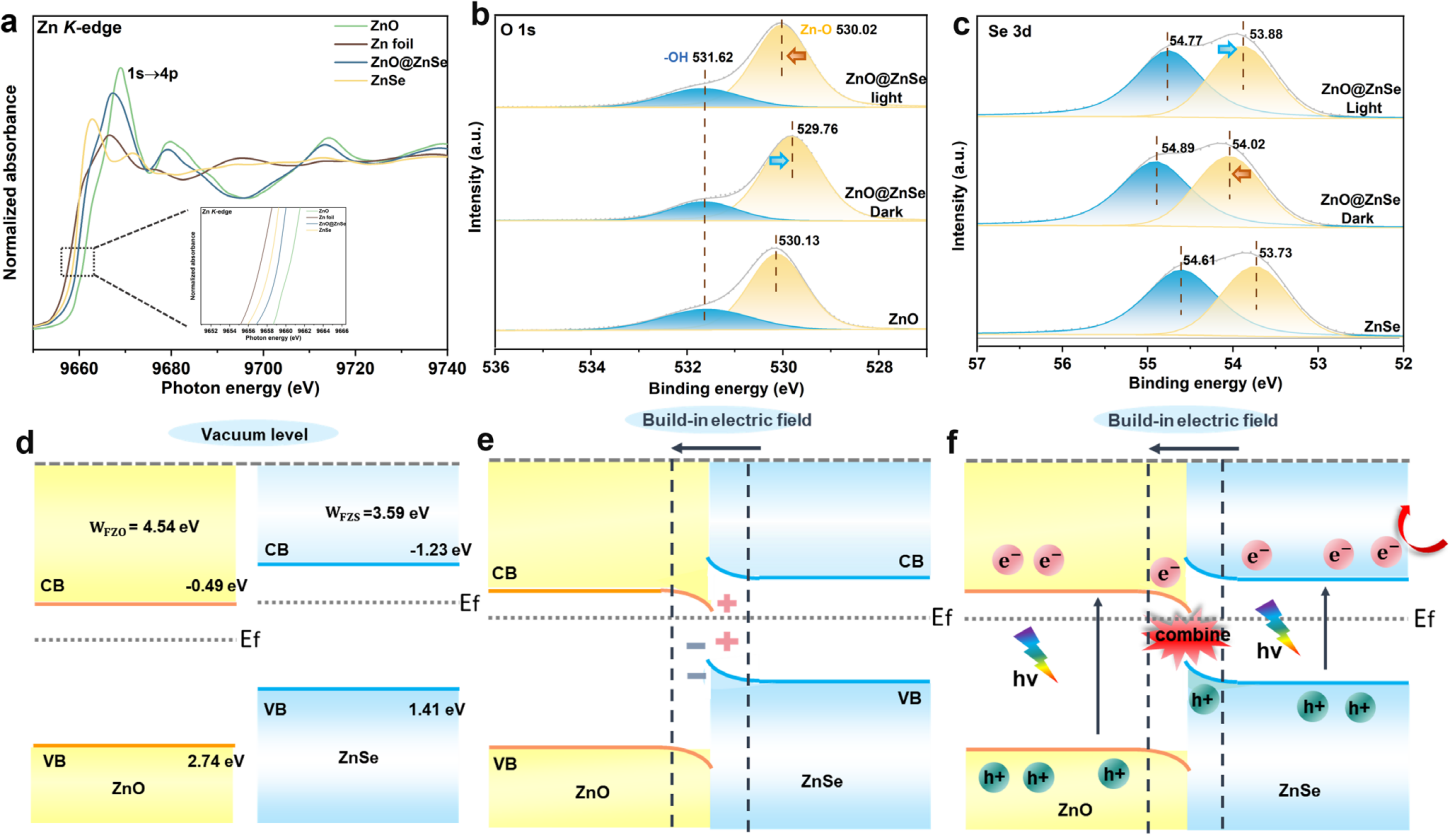
a) Zn K‐edge XANES profiles. b) High‐resolution XPS spectra of O‐1s orbitals. c) High‐resolution XPS spectra of Se‐3d orbitals. d–f) Band structure of ZnO@ZnSe heterojunctions before contact, after contact, and under photoexcitation.

As illustrated in **Figure** **2**a, the Zn K‐edge X‐ray absorption near‐edge structure (XANES) spectrum of the ZnO@ZnSe heterojunction resembles pure ZnO but with intermediate white line intensity between ZnO and ZnSe. The white line intensity directly correlates the electronic transition probability of Zn 1s to 4p states, making this spectral feature an indicator for analyzing the coordination environment around Zn centers through monitoring the occupancy of Zn 4p orbitals. Upon ZnO@ZnSe heterojunction formation, the substitution of interfacial O atoms by Se creates a mixed O‐Zn‐O(Se) coordination environment that integrates the highly localized Zn 4p orbitals from ionic Zn‐O bonds with the partially delocalized Zn 4p orbitals from tendentially covalent Zn‐Se interactions.^[^ ^26^ ^]^ This hybridization causes the Zn 4p orbitals to transition from nearly empty in pure ZnO to partially occupied, thereby reducing the 1s to 4p states' electronic transition probability and resulting in a white line peak intensity that is intermediate between those of pure ZnO and ZnSe. Furthermore, the energy position of near‐edge absorption of Zn in ZnO@ZnSe is located between those of pure ZnO and ZnSe, which can be attributed to electron transfer and redistribution induced by the Fermi level difference at the heterojunction.^[^ ^27^ ^]^

The interfacial charge transfer mechanism of ZnO@ZnSe was clarified through in situ XPS analysis. Ar**^+^** etching‐assisted XPS was employed to analyze Zn, which is distributed across the heterojunction's shell‐core structure. An increase in etching depth revealed the ZnO phase, leading to a positive shift in the Zn 2p binding energy (Figure S7, Supporting Information), which aligns with the XANES data. The O 1s spectrum (Figure 2b) shows characteristic peaks at 530.13 eV (ZnO lattice oxygen) and 531.62 eV (surface ─OH),^[^ ^28^ ^]^ while Se 3d peaks at 54.61 and 53.73 eV correspond to Se^2^^−^ 3d_5/2_ and 3d_3/2_ orbitals in ZnSe.^[^ ^29^ ^]^ After the formation of a heterojunction, the alignment of Fermi levels induces opposing binding energy shifts: the binding energy of lattice oxygen decreases (electron accumulation in ZnO) while the binding energy of Se^2^^−^ increases (electron depletion in ZnSe), establishing a built‐in electric field directed from ZnSe to ZnO. This creates a potential gradient, causing downward band bending near ZnO and upward bending near ZnSe. After irradiating the samples with full‐spectrum light for 20 min, in situ XPS measurements were performed. The peak of lattice oxygen shifts positively (529.76 to 530.02 eV) confirms ZnO electron depletion, while Se^2^^−^ 3d_5/2_ and 3d_3/2_ shifts negatively (54.89/54.02 to 54.77/53.88 eV) verify ZnSe hole depletion. This suggests that the photogenerated electrons of ZnO and the photogenerated holes of ZnSe recombine at the interface.^[^ ^30^ ^]^ The in situ XPS results confirm the successful establishment of the built‐in electric field and demonstrate that this field enhances the separation and transfer of carriers, which is consistent with the typical charge transfer path of S‐scheme heterojunctions.^[^ ^31^ ^]^

To further confirm the successful establishment of the S‐scheme heterojunction, UV–vis, UPS, and VB‐XPS were performed (Figure S8, Supporting Information). The detailed calculation formula can be found in the Supporting Information. On the basis of the Kubelka‐Munk formula, the band gap (*E*g) of ZnO and ZnSe is determined to be 3.23 and 2.64 eV (Figure S8a,b, Supporting Information). VB‐XPS analysis (Figure S8d, Supporting Information) positions the valence bands of ZnO and ZnSe at 2.74 and 1.41 eV (vs NHE), respectively, yielding conduction bands at −0.49 and −1.23 eV when combined with their band gap energies. Based on the secondary electron cut‐off position obtained from the UPS measurements of ZnO and ZnSe (Figure S8c, Supporting Information), the Fermi energy levels of ZnO and ZnSe were determined to be 0.1 and 0.85 eV.^[^ ^32^ ^]^ Given that both ZnO and ZnSe are n‐type semiconductors with interlaced band structures and differing Fermi levels, they satisfy the criteria for forming an S‐Scheme heterojunction (Figure 2d). Combined with the in situ XPS characterization, we successfully constructed a ZnO@ZnSe S‐scheme heterojunction, whose band alignment and charge transfer mechanism are clearly illustrated in Figure 2d‐f.

### 2.3 Investigating Carrier Dynamics and Piezoelectric Field Modulation

**Figure 3:**
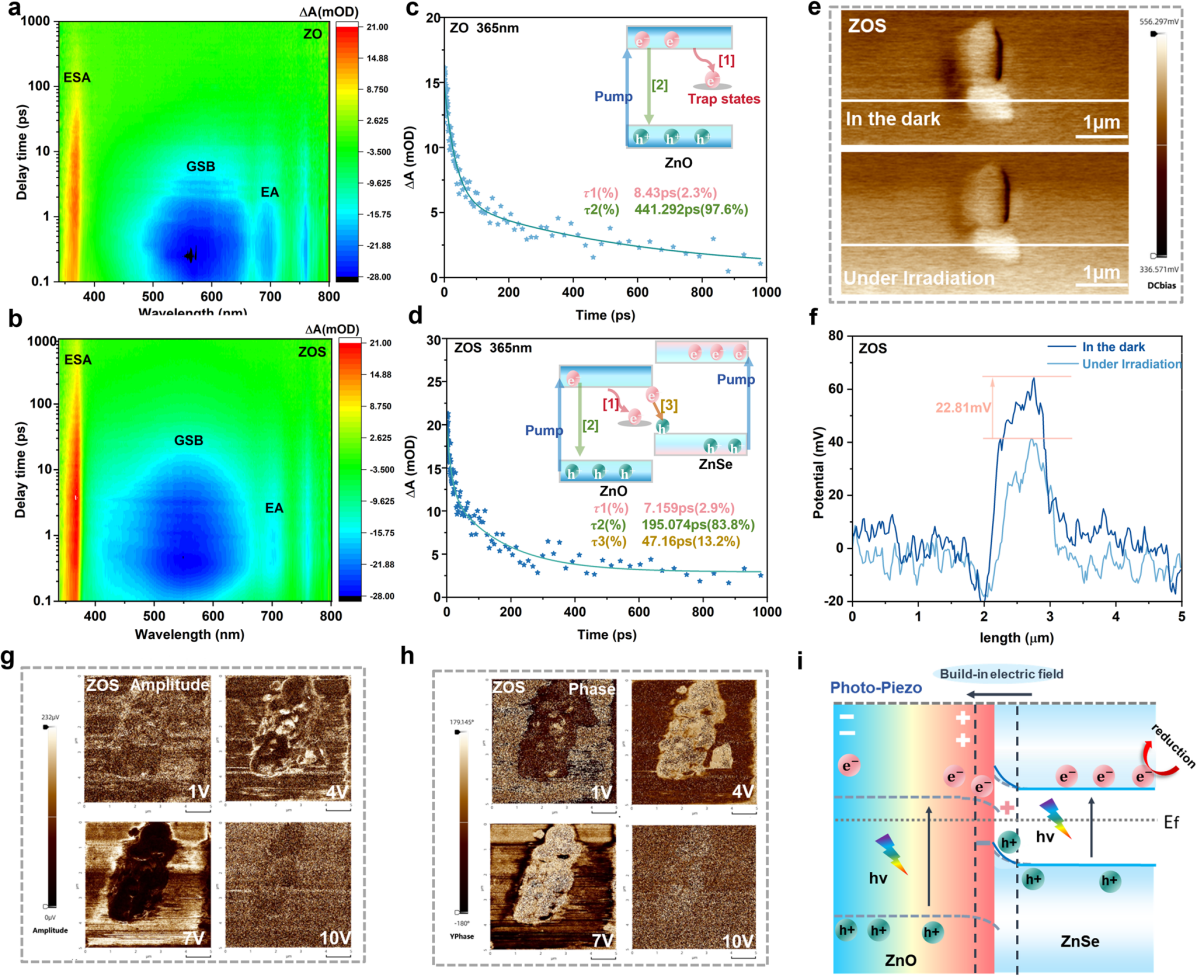
a,b) 3D pseudo‐color spectra of ZO and ZOS acquired via femtosecond transient absorption (fs‐TA) spectroscopy. c,d) Kinetic attenuation curves of ZO and ZOS at 365 nm. e,f) The surface potential of the ZOS catalyst under light and dark conditions was measured using KPFM. g,h) The amplitude and phase of the ZOS samples under various bias voltages were characterized by PFM. i) Mechanism diagram illustrating the regulation of photogenerated carrier behavior by the piezoelectric polarization field.

To gain comprehensive insights into the transportation dynamics in the ZnO@ZnSe S‐scheme heterojunction on a more precise time scale, femtosecond transient absorption (fs‐TA) spectroscopy was further explored. For convenience of description, ZnO, ZnSe and ZnO@ZnSe are abbreviated as ZO, ZS, and ZOS, respectively. The 3D contour line spectra of the fs‐TA spectra for ZO and ZOS are presented in **Figure** **3**a,b. Under 300 nm excitation, both pristine ZO and the ZOS heterojunction exhibited a strong positive signal at 340 to 380 nm, attributed to excited‐state absorption (ESA), reflecting the relaxation dynamics of photoexcited electrons. A pronounced negative signal appeared at 400 to 650 nm, attributed to ground‐state bleaching (GSB), reflecting the depletion of ground‐state carriers due to high‐energy photoexcitation. Additionally, a relatively weak negative signal was observed in the range of 650 to 800 nm, which can be ascribed to stimulated emission (SE).^[^ ^33^ ^]^ As shown in Figure 3a,b, the ZOS S‐scheme heterostructure exhibits significantly enhanced excited‐state absorption (ESA) and ground‐state bleaching (GSB) compared to pristine ZO, demonstrating that the successful formation of the S‐scheme heterojunction promotes charge separation efficiency and substantially increases photogenerated electron density.^[^ ^34^ ^]^ To better understand carrier relaxation in the S‐scheme heterojunction, we analyzed 2D ultrafast spectra (Figure S9a,b, Supporting Information). Compared to pristine ZO, the ZOS heterostructure shows stronger ESA signal variation: ZO's ESA decays within 50 ps, while ZOS maintains a significant signal (≈5 ps) even after 1 ns. This long‐lived excited state electron can be reasonably attributed to the electrons retained on ZnSe VB after the formation of the S‐scheme heterojunction.^[^ ^35^ ^]^

The decay kinetics of the ESA signal at 360 nm revealed different charge carrier relaxation pathways in ZO and ZOS (Figure 3c,d). ZO followed a double‐exponential decay (Figure 3c; Table S3, Supporting Information), and previous studies have confirmed that the shorter lifetime *τ*_1_ represents shallow defect capture, while the longer lifetime *τ*_2_ corresponds to electron‐hole recombination.^[^ ^36^ ^]^ In situ XPS confirms interfacial electron transfer in the ZOS S‐scheme heterojunction, necessitating triexponential decay fitting (Figure 3d; Table S3, Supporting Information). Notably, there is minimal difference between *τ*_1_ of ZOS and pure ZO; however, *τ*_2_ in ZOS is significantly reduced, implying fewer electrons participate in the photogenerated carrier recombination process. This observation indicates that a significant portion of photogenerated electrons rapidly transfers from ZnO to ZnSe upon excitation, thereby shortening the time and proportion of *τ*_2_. Thus, the newly fitted *τ*_3_ in the ZOS decay curve can be reasonably attributed to charge transfer at the interface of the S‐scheme heterojunction.^[^ ^37^ ^]^ In conclusion, upon the formation of the S‐scheme heterojunction, an ultrafast carrier transport pathway is established across the interfaces. This is consistent with the trend obtained from the results of the TPRL and PL tests. (Figure S10, Supporting Information). This approach promotes the dynamic process of photogenerated carriers' separation and migration, thereby enabling more electrons to actively participate in the NO_3_^−^ reduction reaction.

The piezoelectric response force microscopy (PFM) measurement results show that under bias voltages of 1, 4, 7, and 10 V, both the amplitude and phase of ZnO and ZnO@ZnSe materials undergo varying degrees of change (Figure 3g,h; Figure S11, Supporting Information), with both exhibiting clear piezoelectric butterfly curves and phase hysteresis loops (Figure S12, Supporting Information).^[^ ^38^ ^]^ The surface potential of the ZOS sample under both dark and light conditions was systematically investigated using Kelvin probe force microscopy (KPFM) (Figure 3e,f). Upon mechanical stimulation induced by the knocking of the surface probe, a positive surface potential of ≈64.6 mV was observed on the ZOS surface, further corroborating the piezoelectric nature of the ZOS catalyst. Under the illumination condition at 365 nm, the surface potential of ZOS decreased to 41.4 mV, representing a substantial reduction compared to that under dark conditions. This phenomenon can be ascribed to the synergistic effect of the piezoelectric field and the S‐scheme heterojunction. Specifically, the piezoelectric field induced by probe impact modulates photogenerated carrier behavior in the S‐scheme heterojunction, facilitating enhanced electron migration to the surface, where they neutralize positively polarized charges, ultimately reducing surface potential.^[^ ^39^ ^]^ Transient photocurrent response (TPR) measurements reveal that the photocurrent increased by approx≈10 µA cm^−^^2^ under the piezoelectric field, demonstrating improved charge carrier separation and migration kinetics (Figure S13a, Supporting Information).^[^ ^40^ ^]^ Electrochemical impedance spectroscopy (EIS) analysis shows a significantly reduced Nyquist plot radius in the ZOS heterojunction (Figure S13b, Supporting Information), indicating enhanced interfacial electron transfer capability that is further amplified by the piezoelectric polarization field.^[^ ^41^ ^]^

The regulatory mechanism of the piezoelectric field on photogenerated carriers in the ZOS heterojunction is illustrated in Figure 3i. Upon formation of the ZOS S‐scheme heterojunction, interfacial energy band bending creates an ultrafast charge‐transfer pathway that enhances photogenerated electron separation and migration. When the piezoelectric field is introduced, the electron potential is modulated, leading to increased band bending, accelerating the recombination kinetics of photogenerated electrons and holes at the interface, improving carrier separation efficiency.

### 2.4 Piezo‐Photocatalytic Nitrate Reduction to Ammonia: Performance Comparison of Different Catalysts

**Figure 4:**
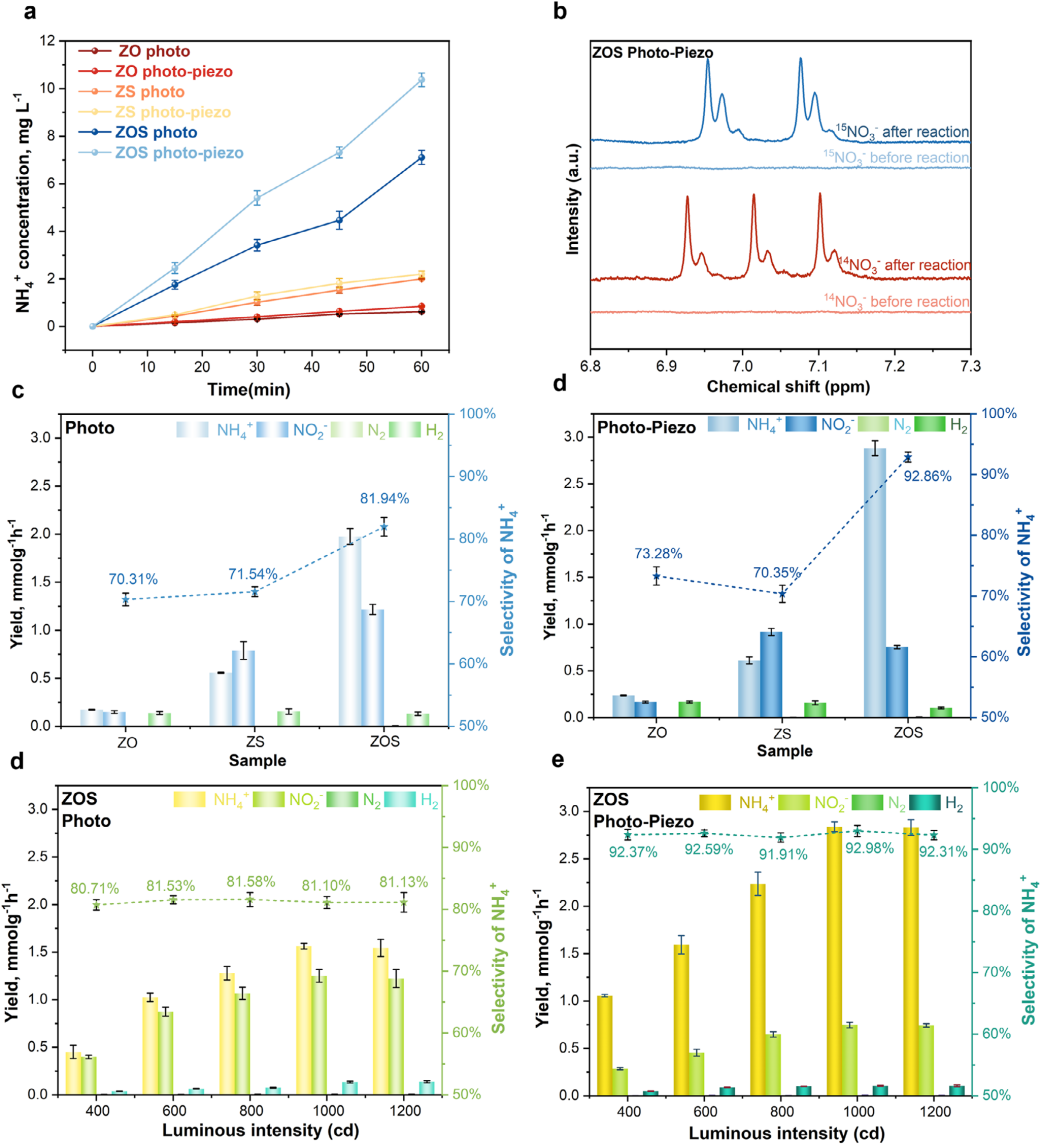
a) NH_4_^+^ synthesis rates of ZO, ZS, and ZOS catalysts under light and ultrasonic‐assisted light conditions. b) Isotopic labeling studies on the nitrogen source in ZOS‐Catalyzed piezo‐photocatalysis. c,d) Yield and NH_4_^+^ selectivity of the products catalyzed by ZO, ZS, and ZOS under light and ultrasonic‐assisted light conditions. e,f) The impact of ultrasound application on the growth rates of NH_4_^+^ production for each catalyst under varying light intensities, as well as NH_4_^+^ production and selectivity under both light and ultrasonic‐assisted light conditions.

Prior to performance testing, HCOONa was selected as the optimal sacrificial agent, establishing the basis for all subsequent experiments (Figure S14, Supporting Information). The NH_4_^+^ production from NO_3_^−^ reduction was evaluated using ZnO, ZnSe, and ZnO@ZnSe (denoted ZO, ZS, ZOS) under both light‐only and ultrasound‐assisted light conditions. NH_4_^+^ concentration was quantified via the indophenol blue method and ^1^H NMR analysis (Figures S15 and S16, Supporting Information). As shown in **Figure** **4**a, ZOS exhibited the highest NH_4_^+^ synthesis rate. Importantly, ^15^N isotopic labeling confirmed the exclusive NO_3_^−^ origin of the produced NH_4_^+^ (Figure 4b). Under light irradiation, ZOS achieved an NH_4_⁺ synthesis rate of 7.106 mg L^−1^ h^−1^, significantly exceeding ZO and ZS due to the S‐scheme heterojunction enhancing charge separation. With ultrasound assistance, the rate increased by 46% to 10.37 mg L^−1^ h^−1^ (Figure S17, Supporting Information), ranking among the highest in reported photocatalytic NH_4_^+^ production. This phenomenon can be reasonably attributed to the further modulation of the internal electric field by the piezoelectric field, which enhances the carrier separation efficiency based on the S‐scheme heterojunction. Comparative analysis under piezoelectric‐only versus photocatalytic conditions (Figure S18, Supporting Information) confirmed the dominant role of photocatalysis in ZOS. After 24 test cycles, ZOS maintained over 70% initial activity (Figure S19, Supporting Information), demonstrating excellent ultrasonic stability. Furthermore, oxidation half‐reaction analysis revealed approximate equality between hole consumption in oxidation and electron consumption in reduction (Figure S20, Supporting Information), verifying charge balance in the catalytic process.

We have tested the output of various competing products and conducted selective calculations. The calculation formulas are as follows: Selectivity of (NH_4_^+^) = [8Yield (NH_4_^+^)] / [8Yield (NH_4_^+^) + 2Yield (NO_2_^−^) + 2Yield (H_2_) + 5/2Yield (N_2_)].^[^ ^42^ ^]^ By integrating the acquired data into Figure 4c,d, it becomes evident that: in comparison to the condition of light alone (Figure 4c), after incorporating ultrasonic assistance (Figure 4d), the ZOS catalyst demonstrates not only a substantial increase in NH_4_^+^ yield but also an enhancement in NH_4_^+^ selectivity from 81.9% to 92.3%. This phenomenon may be attributed to the introduction of a piezoelectric field, which facilitates the participation of a large number of high‐reduction‐potential electrons in the reaction, or it could be due to lattice strain induced by ultrasonic effects, which modulates the active sites during the reduction process.

To clarify the further role of ultrasound in enhancing the selectivity of NH_4_^+^ products, photocatalytic and piezo‐photocatalytic NO_3_^−^ reduction performance tests were conducted under different light intensities, with the results presented in Figure 4e,f. As the light intensity gradually increases, so does the number of incident photons, leading to a corresponding increase in the number of photogenerated carriers. As depicted in Figure 4e, with the rise in carrier density, the yield of NH_4_^+^ progressively increases, yet the selectivity of NH_4_^+^ consistently remains ≈81%. When ultrasonic drive is additionally applied (Figure 4f), the yield of NH_4_^+^ continues to increase, but the selectivity of NH_4_^+^ consistently stabilizes at ≈93%. These test results indicate that the number of electrons involved in the reduction reaction solely influences the yield of NH_4_^+^, not its selectivity. Consequently, in the piezo‐photocatalytic NO_3_^−^ reduction process, ultrasound enhances NH_4_^+^ yield by generating a piezoelectric field in ZnO, which accelerates charge separation and increases electron supply for the reduction reaction. Simultaneously, ultrasound‐induced lattice strain in the ZnSe shell modulates active sites, improving NH_4_^+^ selectivity.

### 2.5 Lattice Strain Induced by Ultrasound

The deformation of ZnO nanorods induced by mechanical stress, which arises from the rupture of cavitation bubbles under ultrasonic excitation, was quantified using the classical cantilever beam strain formula. The classical strain formula for suspended beams is expressed as follows:

(1) $$\varepsilon =4FL/\pi Er^{3}$$

Among them, the definitions of each physical quantity and their corresponding substituted values are summarized as follows: *ε* represents the deformation, and *F* represents the stress, which can be calculated by multiplying the pressure of 1 × 10^8^ Pa generated from the bursting of cavitation bubbles by the area of the acting surface.^[^ ^43^ ^]^ The value of *F* is ≈4 × 10^−6^ N. *L* represents the length of the nanorods, which, according to the statistical analysis of the SEM image, is determined to be 2 micrometers. *E* represents the Young's modulus of ZnO, with a value of ≈1.4 × 10^11^ Pa. *r* represents the radius of the nanorods, which is statistically estimated to be 110 nm based on the SEM image analysis (Figure S21; Table S4, Supporting Information). After substituting all the values into the corresponding equations, the deformation is calculated to be 5%.

### 2.6 The Modulation of Active Sites via Lattice Strain Induced by Ultrasound

**Figure 5:**
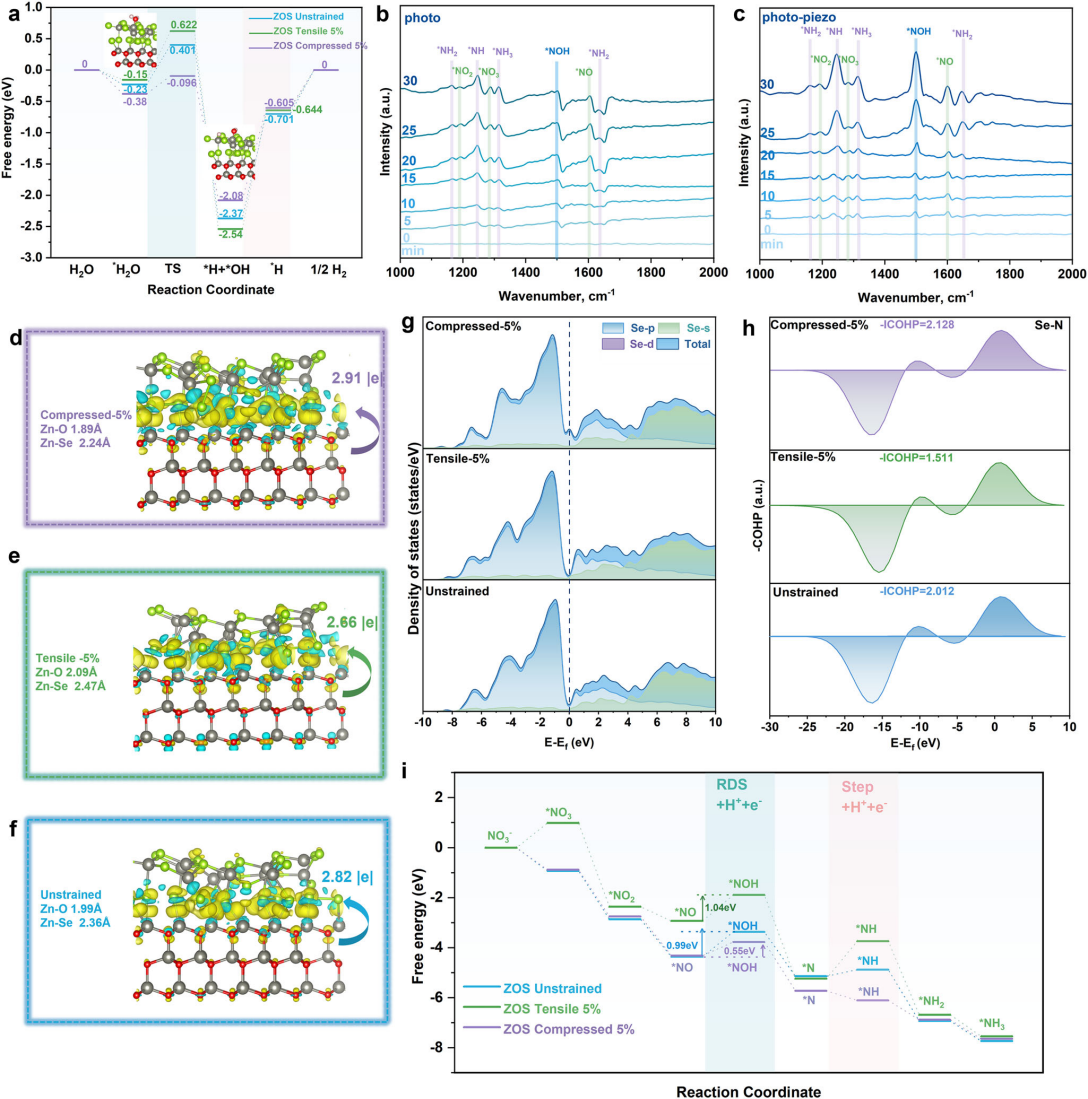
a) Calculated free energy diagram for H_2_O dissociation on the ZOS catalyst in 5% compressed, 5% tensile, and unstrained states. b,c) In situ ATR‐FTIR results for the ZOS photo and piezo‐photocatalytic nitrate‐to‐ammonia. d,f) Charge distribution and Bader charge numbers of the ZOS catalyst in compressed, tensile, and unstrained states. g) electron density of states (DOS) for Se atoms. h) ‐ICOHP values representing the interaction between Se and N(^*^NOH) atoms. i) Gibbs free energy diagrams for NO_3_^−^ reduction on the ZOS catalyst in 5% compressed, 5% tensile, and unstrained states.

Given that the dissociation of H_2_O significantly influences the transfer and coupling processes of hydrogen (^*^H) during NO_3_^−^ reduction, the H_2_O dissociation behavior within the ZnO@ZnSe catalytic system was initially examined. In the reaction solution of photocatalytic NO_3_^−^, a certain amount of *t*‐BuOH, a common active hydrogen quencher, was added to capture the active hydrogen produced during the photocatalytic reaction.^[^ ^44^ ^]^ The results demonstrate (Figure S22, Supporting Information) that the addition of *t*‐BuOH leads to a significant decrease in the NH_4_^+^ yield from the ZnO@ZnSe catalyst, indicating that the ^*^H species generated during H_2_O dissociation play a critical role in the conversion of NO_3_^−^ to NH_4_^+^. Notably, at a *t*‐BuOH concentration of 0.5 mol L^−1^, the reduction in NH_4_^+^ yield is less pronounced under ultrasonic‐assisted conditions compared to that under no ultrasonic‐assisted conditions, suggesting that ultrasound‐induced lattice strain enhances H_2_O dissociation, thereby facilitating greater participation of ^*^H in the NO_3_^−^ reduction process. The dissociation of H_2_O, which spontaneously adsorbs onto Zn atoms, was systematically investigated using density functional theory (DFT) calculations, revealing the Gibbs free energy associated with H_2_O dissociation on ZnO@ZnSe catalysts under unstrained, 5% tensile strain, and 5% compressive strain conditions, as presented in **Figure** **5**a. Under 5% compressive strain, ZnO@ZnSe exhibits the lowest energy barriers for both H_2_O dissociation (0.284 eV) into ^*^H+^*^OH and subsequent ^*^H overflow (1.475 eV), facilitating ^*^H migration to Se sites for efficient NO_3_^−^ reduction.^[^ ^45^ ^]^ The detailed model is shown in Figure S23 (Supporting Information).

The data obtained from in situ Raman spectroscopy indicate that as the reaction time increases, the characteristic peak of NH_4_^+^ becomes more prominent (Figure S24, Supporting Information). In situ ATR‐FTIR results obtained under light conditions and ultrasonic‐assisted conditions are presented in Figure 5b,c. The absorption peaks at 1162 cm^−1^ (1654 cm^−1^) and 1251 cm^−1^, as well as 1316 cm^−1^, correspond to the intermediates ^*^NH_2_, ^*^NH and ^*^NH_3_, respectively, which contain N─H bonds.^[^ ^46^ ^]^ The absorption peaks at 1194, 1282, and 1604 cm^−1^ correspond to the intermediates ^*^NO_2_, ^*^NO_3,_ and ^*^NO, respectively, which contain N═O bonds.^[^ ^47^ ^]^ Comparative analysis of Figure 5b,c reveals markedly enhanced intensities for all intermediate peaks under ultrasonic stimulation. This demonstrates that the piezoelectric field generated by ultrasound enhances carrier separation, thereby increasing electron participation in the reaction and consequently improving the yield of NH_4_^+^. Remarkably, the peak intensity of the key intermediate ^*^NOH (1499 cm^−1^) markedly increases after ultrasonic application.^[^ ^48^ ^]^ This indicates that ultrasonic assistance promotes the transformation of ^*^NO into the critical intermediate ^*^NOH, thereby preventing the accumulation of NO_2_^−^ and facilitating subsequent H‐addition steps. Moreover, no NHOH and NH_2_OH intermediates were detected during in situ ATR‐FTIR analysis, confirming that the NO_3_^−^ reduction pathway for NH_4_^+^ production on the ZOS catalyst proceeds as follows: NO_3_^−^→^*^NO_3_→^*^NO_2_ →^*^NO→^*^NOH→^*^N→^*^NH→^*^NH_2_→^*^NH_3_. At the same time, the results of the DFT calculation also confirm this. The thermodynamic process of NHOH and NH_2_OH on ZnO@ZnSe is unfavorable. Please refer to Figures S25 and S26 (Supporting Information).

To gain deeper insight into how ultrasound‐induced lattice strain modulates the electronic properties of active‐site Se atoms, we performed comprehensive Bader charge analyses and density of states calculations for Se atoms in the ZOS heterojunction under various strain conditions (unstrained, 5% tensile, and 5% compressive), complemented by crystal orbital Hamiltonian population (COHP) analysis to probe the Se‐^*^NOH orbital interactions. Figure 5d,f maps strain‐induced Bader charge transfer at the interface. When ZOS is under compressive strain, the Bader charge transfer at the interface reaches its maximum value of 2.91|e|, indicating that a greater number of electrons are transferred to the surface and actively participate in the reaction. As clearly demonstrated in Figure 5g, the application of 5% compressive strain markedly enhances the electronic density of states near the Fermi level in Se atoms, a modification known to strengthen orbital hybridization with reaction intermediates and promote charge transfer kinetics.^[^ ^49^ ^]^ Therefore, to further explore the orbital interaction between the active site Se and the N in the key intermediate ^*^NOH, the COHP analysis was conducted, and the results are shown in Figure 5h. Upon applying 5% compressive strain, the ‐ICOHP value between Se and N(^*^NOH) atoms in ZOS becomes the largest, indicating a stronger orbital interaction.^[^ ^50^ ^]^ This effectively facilitates the formation of ^*^NOH key intermediates, which is in agreement with the result of in situ ATR‐FTIR.

The comprehensive assessment of lattice strain‐regulated Se active sites' impact on the overall reaction process necessitated Gibbs free energy calculations for the nitrate reduction pathway. The detailed computational model and corresponding results are presented in Figure 5i and Figure S27 (Supporting Information). Upon adsorption of the intermediate on the 5% tensile strain, the transformation from NO_3_^−^ to ^*^NO_3_ requires heat absorption. In contrast, the unstrained and 5% compressed ZOS lattice exhibit exothermic spontaneous reactions during the first step, making the reaction more favorable on the unstrained and 5% compressed side. In the rate‐determining step (^*^NO to ^*^NOH), the 5% compressed ZOS lattice demonstrates a lower energy barrier (△G = 0.55 eV) compared to the unstrained state (△G = 0.99 eV). This suggests that 5% compressive strain facilitates the occurrence of the ^*^NO to ^*^NOH reaction at the active site, thereby accelerating the hydrogenation process and mitigating NO_2_^−^ accumulation. Additionally, during the ^*^H‐addition step (^*^N to ^*^NH), the 5% compressive strain ZOS heterojunction undergoes spontaneous reactions, whereas the unstrained and tensile lattices require overcoming an energy barrier. In summary, the ZnSe shell compressed lattice strain caused by ultrasound increases the density of states of the active‐site Se atoms near the Fermi level, enhances the Se‐N orbital interaction between Se atoms and the critical intermediate ^*^NOH, reduces the energy barrier in the rate‐determining step (^*^NO to ^*^NOH), thereby promoting the addition of ^*^H step and improving the catalytic performance.

## 3 Conclusion

In conclusion, we have successfully developed a piezoelectric S‐scheme ZnO@ZnSe heterojunction with robust interfacial coupling via in situ interface engineering. The introduction of mechanical stimulation through ultrasound generates a piezoelectric field within the material. This field further modulates the band structure of the S‐scheme heterojunction, enhancing charge carrier separation and migration, leading to increased NH_4_⁺ production efficiency. More significantly, ultrasound‐induced mechanical stimulation simultaneously induces lattice strain within the ZnSe shell. This lattice strain accelerates the rate of H_2_O dissociation on the Zn atoms, modulates the electronic state density of the Se atoms, optimizes the thermodynamic pathway of nitrate reduction, and consequently enhances the selectivity of the reaction. As a result, a highly selective and efficient NO_3_^−^ reduction reaction is achieved through the synergistic interplay of the coupled S‐scheme heterojunction, piezoelectric field, and lattice strain. This work provides valuable insights for the rational design of advanced piezoelectric catalysts for multi‐electron catalytic reactions.

## Conflict of Interest

The authors declare no conflict of interest.

## Supporting information



Supporting Information

## Data Availability

The data that support the findings of this study are available from the corresponding author upon reasonable request.;
