# Supplementary material for: Lattice Strain and Piezoelectric Field Modulated Piezo‐Photocatalytic Nitrate‐to‐Ammonia Conversion in Chemically Bonded S‐Scheme Heterojunction
Source: Adv Sci (Weinh). 2025 Nov 19;13(7):e18794. doi: 10.1002/advs.202518794 (PMC12866831; doi:10.1002/advs.202518794)
Supplement: Supplementary file 1 — Supporting Information [file ADVS-13-e18794-s001.docx]

Supporting Information

**Lattice Strain and Piezoelectric Field Modulated Piezo-Photocatalytic** **Nitrate-to-Ammonia Conversion in Chemically Bonded S-Scheme Heterojunction**

Shuo Liu, Jing Li, Zhiwei Liu, Jiaxuan Song, Huijun Lv, Dongdong Xiao, Qizhao Wang*, Yongzheng Zhang*, Qikun Xue

S. Liu, Z. Liu, J. Song, H. Lv, Y. Zhang

School of Physics and Physical Engineering, Qufu Normal University, Qufu 273165, China

E-mail: yzzhang@qfnu.edu.cn

Q. Wang

College of Chemistry and Chemical Engineering, Northwest Normal University, Lanzhou 730070, China

E-mail: qzwang@chd.edu.cn

J. Li

Technical Institute of Physics and Chemistry, Chinese Academy of Sciences, Beijing 100190, China

Y. Zhang, Q. Xue

Quantum Science Center of Guangdong-Hong Kong-Macao Greater Bay Area, Shenzhen 518102, China

D. Xiao

Institute of Physics Chinese Academy of Sciences, Beijing 100190, China

**Experiment Section**

1. **Chemicals**

All the reagents were of analytical grade and used without additional treatment. Zinc nitrate hexahydrate(Zn(NO_3_)_2_·H_2_O), hexamethylenediamine(HMTA), selenium powder(Se), sodium borohydride(NaBH_4_), sodium hydroxide(NaOH), zinc acetate dihydrate(Zn(CH3COO)_2_·H_2_O), diethanolamine(DEA), Sodium salicylate(C_7_H_5_O_3_Na,), sodium hypochlorite(NaClO), sodium nitrosyroferricyanide dehydrate(C_5_FeN_6_Na_2_O), ammonium chloride(NH_4_Cl), sulfonamid (C_6_H_8_N_2_O_2_S), naphthylenediamine hydrochloride (C_12_H_14_N_2_·2HCl), sulfamic acid, Hydrochloric acid, phosphoric acid(H_3_PO_4_), sodium formate(HCOONa), potassium nitrate(KNO_3_), potassium nitrite(KNO_2_), Potassium nitrate K^15^NO_3_ (Aladdin 99atom%), Anhydrous cerium(IV) sulfate(Ce(SO_4_)_2_), Maleic acid(C_4_H_4_O_4_).

1. **Methods**

*Synthesis of ZnO Nanoarrays*: By employing the magnetron sputtering growth of a ZnO seed layer combined with the hydrothermal method, a ZnO nanorod array with uniform morphology and excellent transverse and longitudinal properties was successfully fabricated.^[1]^ The detailed experimental procedures are as follows: (i) ITO glass substrates (2.5 cm × 1 cm) were ultrasonically cleaned sequentially in acetone, ethanol, and deionized water for effective surface preparation, followed by drying at room temperature for subsequent use. (ii) A radio frequency magnetron sputtering system was utilized to deposit the ZnO seed layer under an Ar gas flow rate of 20 sccm and a power setting of 35 W for 5 minutes. Subsequently, the coated ITO glass was annealed in a tube furnace at 200 °C for 20 minutes to stabilize the seed layer. (iii) The ITO glass with the deposited seed layer was immersed in a mixed solution containing 50 mM of zinc nitrate hexahydrate (Zn (NO_3_)_2_·6H_2_O) and 50 mM of hexamethylenetetramine (HMTA) dissolved in 50 ml of deionized water. The solution was heated in a water bath at 85 °C for 18 hours. After completion, the sample was thoroughly washed with anhydrous ethanol and deionized water and dried in a vacuum oven at 60 °C overnight.

*Heteroepitaxial Growth of ZnO@ZnSe Core-Shell Structure*: In this experiment, 0.03 g of selenium powder (Se) and 0.1 g of sodium borohydride (NaBH_4_) were dissolved in 50 ml of deionized water. Argon gas was introduced to remove the dissolved oxygen from the solution, which was subsequently heated to 60 °C. An ITO glass substrate with ZnO nanorod arrays was immersed in the solution.^[2]^ After a reaction time of 2 hours, the ITO glass was removed, ultrasonically cleaned with ethanol and deionized water, and then vacuum-dried at 60°C overnight.

*Synthesis of Pure ZnSe*: First, selenium powder, zinc acetate dihydrate (C_4_H_10_O_6_Zn), and sodium hydroxide (NaOH) were accurately weighed in a molar ratio of 1:1:8 (1.5792 g, 4.3902 g, and 6.4 g, respectively) and subsequently dissolved in 60 mL of deionized water contained in separate beakers. The solutions were then stirred continuously for 1 hour to ensure complete dissolution ^[3]^. Next, 6 mL of diethanolamine (DEA) was added to the mixture, followed by additional stirring for 30 minutes. Subsequently, the resultant solution was transferred into a 100 mL Teflon-lined stainless steel autoclave and heated at 200 °C for 24 hours under autogenous pressure. Upon completion of the reaction, the autoclave was allowed to cool naturally to room temperature. The resulting precipitate was collected, washed thoroughly with ethanol and deionized water three times to remove any residual impurities, and finally dried overnight at 60 °C in a vacuum oven.

1. **Material Characterization**

SEM images were acquired via scanning electron microscopy (SEM, Carl Zeiss Ultra 55, 5kV). The phase information of the sample was characterized using X-ray diffraction analysis (XRD,Rigaku D/Max-cA, Japan). High-angle annular dark-field microscopy was employed to determine the lattice structure and atomic arrangement of the sample (HAADF-STEM, JEM-2100F, JEOL, Japan). The band structure was systematically analyzed through ultraviolet photoelectron spectroscopy (UPS, AXIS SUPRA+, SHIMADZU, UK) and Ultraviolet-visible diffuse reflection spectra (UV-Vis DRS, Lambda 1050, PerkinElmer, USA). The dynamics and lifetime of photogenerated carriers were investigated by X-ray photoelectron spectroscopy (XPS, AXIS SUPRA+, SHIMADZU, UK) and femtosecond transient absorption spectroscopy(fs-TA). Carrier lifetime was quantitatively evaluated using photoluminescence (PL, Edinburgh, FLS1000, UK) and time-resolved photoluminescence (TRPL, Edinburgh, FLS1000, UK) techniques. Photoelectric performance of the samples was assessed by photocurrent and impedance measurements conducted on the CHI-760e electrochemical workstation. The influence of piezoelectric polarization electric fields on carrier behavior was explored using Kelvin probe force microscopy (KPFM, AtomEdge Pro) and piezoelectric force microscopy (PFM, AtomEdge Pro). In-situ ATR-FTIR (Bruker VERTEX 80V) and In-situ Raman (Horiba LabRAM HR Evolution, 532nm) spectroscopy were utilized to investigate the behavior of intermediates, while first-principles calculations elucidated (DFT) the underlying reaction mechanism.

1. **The X-ray absorption fine structure (XAFS) tests**

X-ray absorption fine structure (XAFS) spectroscopy was carried out using the Deep-Inspectra-X1(Beijing SciStar Technology Co., Ltd.) by transmission mode at 20 kV and 40 mA, and the Si (800) spherically bent crystal analyzer with a radius of curvature of 500 mm was used for Zn. The Se K-edge absorption spectrum was recorded at 12658 eV at the BL14B2 beamline of the SPring-8 synchrotron light source. The beam was monochromatized using a (311) or (111) double-crystal monochromator and focused through a rhodium-coated mirror, achieving a standard beam size of 5 mm horizontally and 0.5 mm vertically.

1. **Evaluation of Photocatalytic Activity**

In the customized photocatalytic reactor, 10 mg of the catalyst was added to 50 mL of KNO_3_ solution with a NO_3_^-^ concentration of 100 mg L^-1^. Subsequently, 0.816 g of sodium formate was introduced to achieve a sacrificial agent concentration of 0.25 mM. Prior to initiating the reaction, Ar gas was purged into the system for 30 minutes to remove dissolved oxygen and nitrogen from the solution. During this degassing process, the system was kept in complete darkness to prevent any unintended photochemical reactions. Following the degassing step, the reactor was sealed, condensate water was circulated, and the system was placed in an ultrasonic bath operating at 480 W. The experimental setup is illustrated in the subsequent diagram. Piezo-photocatalytic nitrate reduction was performed under ultrasound irradiation and illumination by a 300 W xenon lamp. Samples are collected every 15 minutes for yield analysis. Both nitrogen and hydrogen are sampled using the needle injection method.

1. **Determination of Product Concentration**

*Determination of H_2_ and N_2_*

All gaseous products were detected by gas chromatography. Both nitrogen and hydrogen are sampled using the needle injection method.

*Determination of NO_3_^-^*

Take a specific reaction solution and dilute it to within the 5 mL detection range. Subsequently, add 0.1 mL of 1 M HCl solution and 0.01 mL of 0.8 wt% sulfamic acid solution to the diluted solution. Allow the mixture to stand for 20 minutes before conducting the analysis using a UV-Vis spectrophotometer. Record the absorbance values of the solution at wavelengths of 220 nm and 270 nm. The final absorbance is calculated as A(NO_3_^-^) = A (220 nm) - 2A (270 nm). By preparing a series of NO_3_^-^ standard solutions with varying concentrations, a concentration-absorbance calibration curve for NO_3_^-^ can be constructed.

*Determination of NO_2_^-^*

Dissolve 4 g of sulfonamid (C_6_H_8_N_2_O_2_S), 0.2 g of naphthylenediamine hydrochloride (C_12_H_14_N_2_·2HCl), and 10 mL of phosphoric acid (H_3_PO_4_) in 50 mL of deionized water to prepare the chromogenic reagent. Pipette 5 mL of the sample solution into a clean cuvette, add 0.1 mL of the prepared chromogenic reagent, mix thoroughly, and allow it to stand for 20 minutes at room temperature. Subsequently, measure the absorbance at 540 nm using a UV-Vis spectrophotometer. To construct the calibration curve, prepare a series of NO_2_^-^ standard solutions with varying concentrations, determine their absorbances under identical conditions, and plot the concentration-absorbance relationship.

*Determination of NH_4_^+^*

Ammonia was quantified using indole blue spectrophotometry (IBS). To prepare the chromogenic reagents, dissolve 0.2 g of sodium nitrosyl ferricyanide (C_5_FeN_6_Na_2_O) in 20 mL of deionized water (DI) to form chromogenic reagent A. For chromogenic reagent B, dissolve 6.404 g of sodium salicylate (C_7_H_5_O_3_Na) and 1.312 g of sodium hydroxide (NaOH) in 100 mL of DI. Chromogenic reagent C is prepared by dissolving 1.5 mL of sodium hypochlorite (NaClO) and 0.615 g of sodium hydroxide (NaOH) in 20 mL of DI. Subsequently, take 4 mL of the sample solution and sequentially add 2.4 mL of reagent B, 800 μL of reagent C, and 320 μL of reagent A. Allow the mixture to stand at room temperature in the dark for 2 hours before measuring its absorbance at 655 nm using a UV-visible spectrophotometer. By preparing a series of NH_4_^+^ standard solutions with varying concentrations, a calibration curve correlating concentration to absorbance can be established.

*Determination of H_2_O_2_*

Based on the proposed mechanism, the production of H₂O₂ was quantitatively determined.

2Ce^4+^ + H_2_O_2_ = 2Ce^3+^ + 2H^+^ + O_2_

*C* _H2O2_ = 1/2△*C*_Ce4+_

First, a series of Ce^4+^ solutions with known concentrations (0.05 mM, 0.1 mM, 0.15 mM, 0.2 mM, and 0.25 mM) were prepared. Their absorbance at 319 nm was measured to establish a concentration-absorbance standard curve. The collected post-reaction solution was then mixed with the 0.25 mM Ce^4+^ solution at a volume ratio of 1:100. After standing for 2 hours, the absorbance of the mixture at 319 nm was measured to determine the remaining concentration of Ce^4+^. The H_2_O_2_ yield was calculated based on the change in Ce^4+^ concentration.

1. **Photoelectrochemical test**

The tests were conducted on an electrochemical workstation (CHI-760E, Shanghai Chenhua Apparatus Corporation, China) using a standard three-electrode system, in which platinum mesh, test samples dropcoated on indium tin oxide (ITO) glass and Ag/AgCl electrode were used as counter electrode, working electrode and reference electrode, respectively. For transient photocurrent response (TPR) tests, the optical source is a 300 W xenon lamp (PLS-SXE 300+, Perfect Light, China) equipped with an UV-enhanced reflection sheet and the electrolyte is the Na_2_SO_4_ solution with a concentration of 0.5 mol/L. The electrolyte used for electrochemical impedance spectroscopy (EIS) measurements (the frequency signal of 100 kHz to 0.01 Hz) consists of a mixture of 5 mmol/ L K_3_[Fe (CN)_6_]/K_4_[Fe (CN)_6_] and 0.5 mol/L KCl, and the signals are recorded from 100 kHz to 0.01 Hz. During the piezo-photoelectrochemical test, the three-electrode electrolytic cell used was fixed on the iron stand in the ultrasonic machine to apply mechanical stimulation. All the other tests were exactly the same as the photoelectrochemical test.

1. **In situ ATR-FTIR and in situ Raman test**

In situ ATR-FTIR was conducted on Bruker VERTEX 80v.The measurements were all obtained by 120 scans at a spectral resolution of 8 cm^−1^. An ink was prepared by mixing 5 mg of catalyst, 40 μL of Nafion solution, 720 μL of ethanol, and 240 μL of deionized water, and uniformly coated onto a germanium crystal. A certain amount of 100 mg L^-1^ nitrate aqueous solution was injected into the in-situ test cell, and a small vibration motor was immersed below the liquid surface to serve as a mechanical stimulation source. Simultaneously, the germanium crystal was irradiated using a 300W xenon lamp. After applying mechanical stimulation and light irradiation for a predetermined duration, the light source and vibration were turned off, and the test was initiated. To prevent the accumulation of intermediates, fresh ink was coated onto the germanium crystal and the nitrate solution was replaced before each test.

In situ Raman spectroscopy was performed using a confocal Raman microscope (Horiba LabRAM HR Evolution) with a 532 nm single longitudinal-mode laser at room temperature. An ink was prepared by mixing 5 mg of catalyst, 40 μL of Nafion solution, 720 μL of ethanol, and 240 μL of deionized water, and uniformly coated onto carbon paper. The carbon paper was then clamped into an in-situ Raman test cell. A certain amount of 100 mg/L nitrate aqueous solution was added, completely covering the catalyst. A small vibration motor was immersed below the liquid surface to act as a mechanical stimulation source, while a 300W xenon lamp was used to irradiate the carbon paper. After applying mechanical stimulation and light irradiation for a predetermined period, the light source and vibration were turned off, and the testing began.

1. **^1^H NMR Characterization**

A series of ammonium ion standard solutions were prepared at concentrations of 25, 50, 100, 150, 200, and 250 mg L^-1^. The pH of each solution was adjusted to 2–3, followed by the addition of a precise amount of maleic acid to achieve a final concentration of 0.4 mg mL^-1^. For NMR analysis, 0.45 mL of the prepared solution was mixed with 0.05 mL of D₂O and analyzed using a 600 MHz NMR spectrometer. The standard curve was constructed by plotting the ratio of the ¹H peak area (of ammonium ions) to that of the internal standard (maleic acid with known concentration) on the y-axis, against the NH₄⁺ concentration on the x-axis.

For quantitative analysis: Following the catalytic reaction, the solution was acidified with 0.1 M H₂SO₄ to adjust the pH to 2–3. A predetermined amount of maleic acid was then added to achieve a final concentration of 0.4 mg mL^-1^. Subsequently, 0.45 mL of this treated solution was mixed with 0.05 mL of D₂O and analyzed by 600 MHz NMR. The measured peak area ratio of the analyte to the internal standard (maleic acid) was then applied to the standard curve to determine the ammonia concentration.

In the isotopic labeling experiment to determine the source of ammonia, KNO₃ was replaced with an equivalent mass of K¹⁵NO₃ (99 atom% ¹⁵N), while all other test conditions were kept consistent with those used for K¹⁴NO₃.

1. **fs-TA test**

Transient absorption (TA) spectroscopy was carried out by using a Helios pump-probe system (Ultrafast Systems) combined with a regenerative-amplified Ti: sapphire laser system with the central wavelength of 800 nm, the pulse duration of 25 fs and the repetition rate of 1 kHz (Legend Elite-1K-HE, Coherent). The output light of regenerative-amplified Ti: sapphire laser with central wavelength of 800 nm was split into two beams. The main part of the fundamental beam (800 nm) was sent to the synchronized optical parametric amplifiers (TOPAS-C), which generated a pump pulse with wavelength of 300 nm. A small part of the fundamental beam (800 nm) was introduced into the TA spectrometer in order to generate the probe light. The motorized optical delay-line precisely controlled the optical path difference between the pump and probe beams, enabling accurate measurement of their relative time delay. After passing through a motorized optical delay line, the probe beam was attenuated with a neutral density filter and focused on a CaF_2_ crystal, which was used to generate the white-light continuum pulses with wavelength of 300 to 800 nm. The probe beam and the pump beam were focused and overlapped onto the sample. After sampling, the probe beam was collimated and then coupled into a fiber-coupled spectrometer, which was detected at a frequency of 1 KHz. A synchronized chopper operating at frequency of 500 Hz modulated the pump pulses, enabling alternate recording of TA spectra with and without pump excitation. The intensity of the pump pulse used in the experiment was controlled by a variable neutral-density filter wheel. All experiments were performed at room temperature. The analysis was carried out by using Surface Xplorer software.

**Theoretical section**

**First-principles calculation method**

All the calculations were carried out with density functional theory (DFT) method as implemented in the Vienna Ab Initio Simulation Package (VASP). The electron ion interaction was described with the projector augmented wave (PAW) method, while the electron exchange and correlation energy were solved within the generalized gradient approximation with the Perdew-Burke-Ernzerhof (PBE) exchange-correlation functional unless stated otherwise. The kinetic energy cutoff of plane wave was set to be 450 eV and the convergence criterion for the residual forces and total energies were set to be 0.03 eV/Å and 10^-5^ eV, respectively.^[4]^

The empirical correction in Grimme’s method (DFT+D3) was adopted to describe van der Waals interaction. In this study, the supercell model is defined with lattice parameters of a =14.15 Å, b =14.15 Å, c = 22.68 Å, and α = β = γ = 90°. The vacuum space in the z direction was set to be 14 Å, which was large enough to minimize the interaction between periodic images, and a 1x1x1 Monkhorst-Pack k-point mesh was used to sample the Brillouin zone. The reaction Gibbs free energy ∆G is defined by the following equation:

∆G = ∆E + ∆E_ZPE_ - T∆S (3)

where ΔE is the binding energy, ΔE_ZPE_ is the change in zero-point vibrational energies, T is the temperature, and ΔS is the entropy change. The ideal gas approximation and the harmonic approximation were used, and for adsorbates all atomic nuclear motions were considered as harmoni oscillators.

**Supplementary Figures**

**
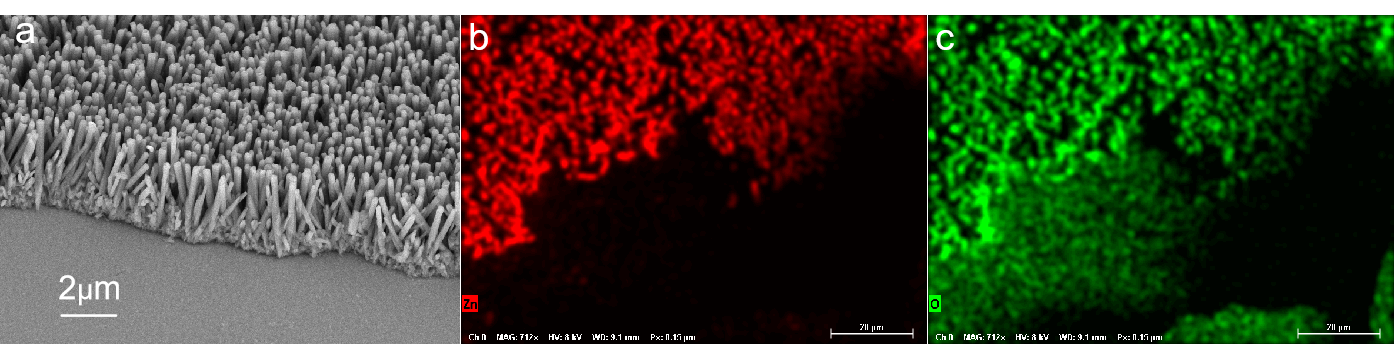
**

**Figure S1**. SEM and EDS images of ZnO nanorods array

**
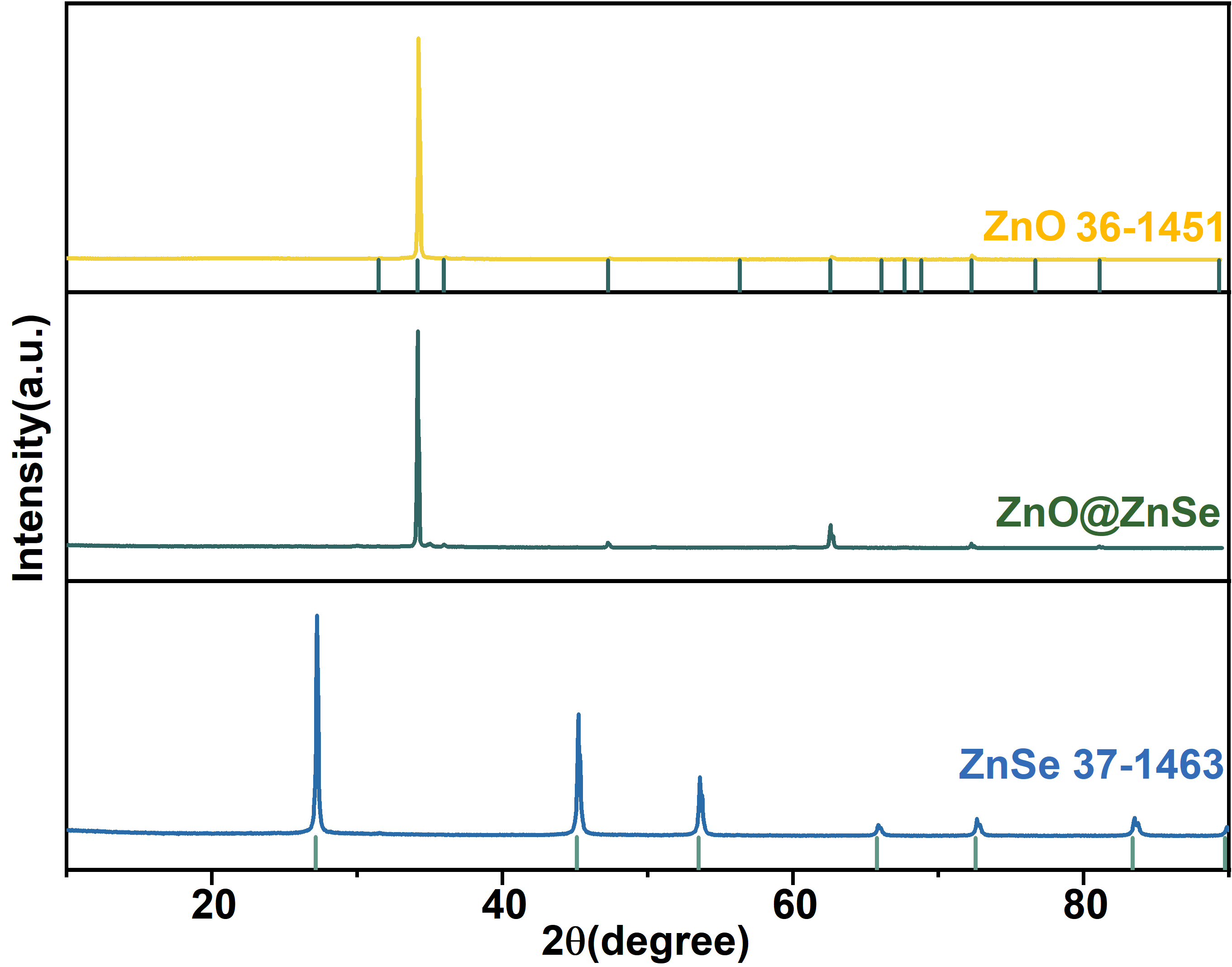
**

**Figure S2**. XRD image of ZnO, ZnSe, ZnO@ZnSe.

It should be noted that due to the extremely thin and low content of the ZnSe shell, XRD cannot obtain the diffraction peaks of ZnSe. The existence of ZnSe can be confirmed through XPS and TEM tests.

**
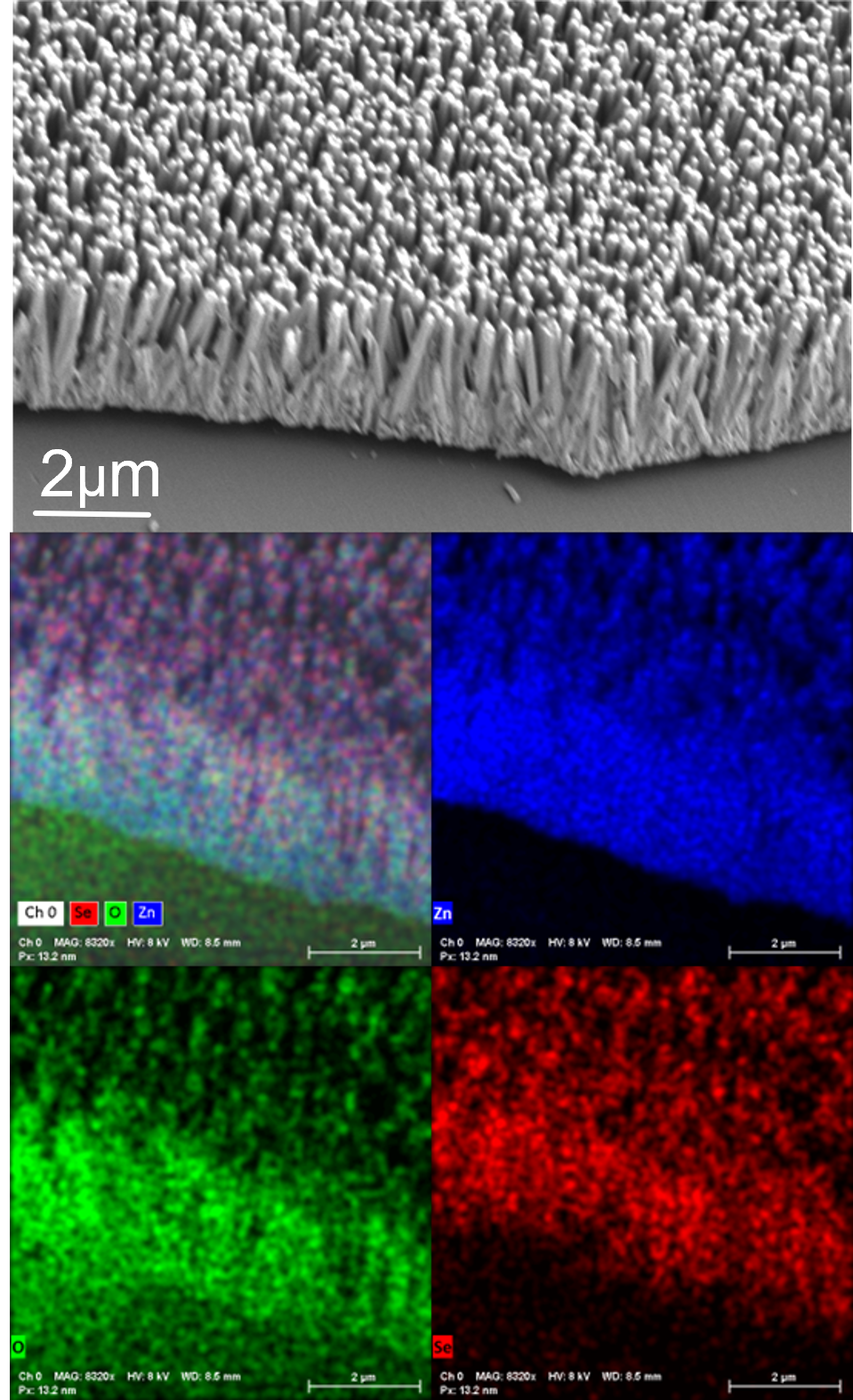
**

**Figure S3.** SEM and EDS image of ZnO@ZnSe nanorods array

**
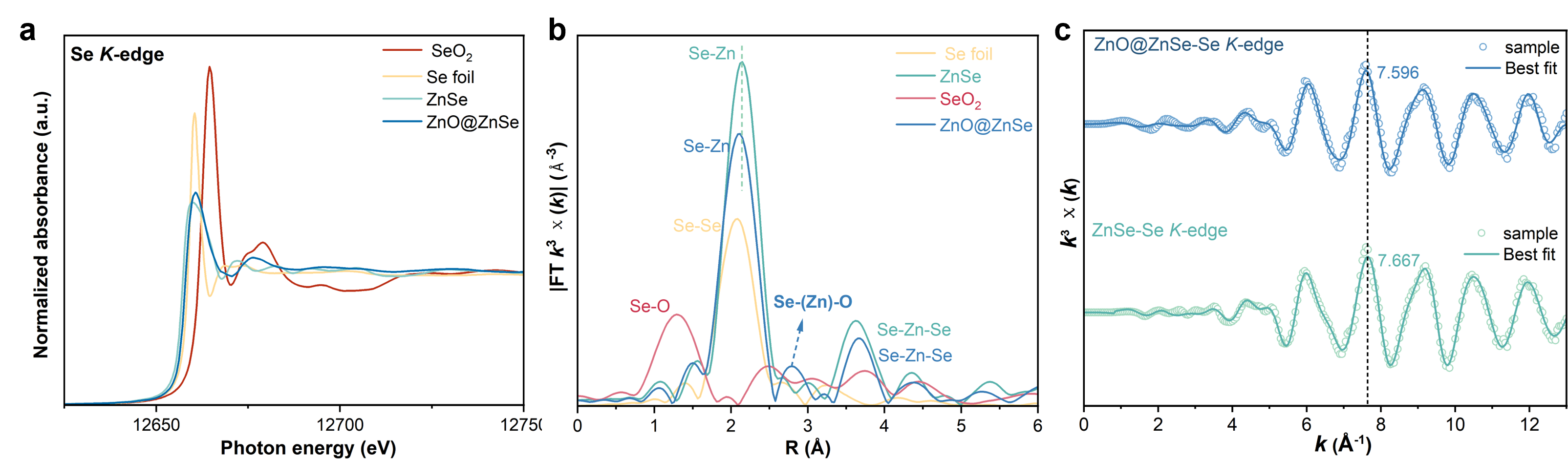
**

**Figure S4.** (a) Normalized Se K-edge XANES spectra. (b) *k*^3^-weighted Fourier-transform magnitudes of the Se K-edge EXAFS spectra. (c) *k*-space EXAFS oscillations and fitting results for the Se K-edge.

**
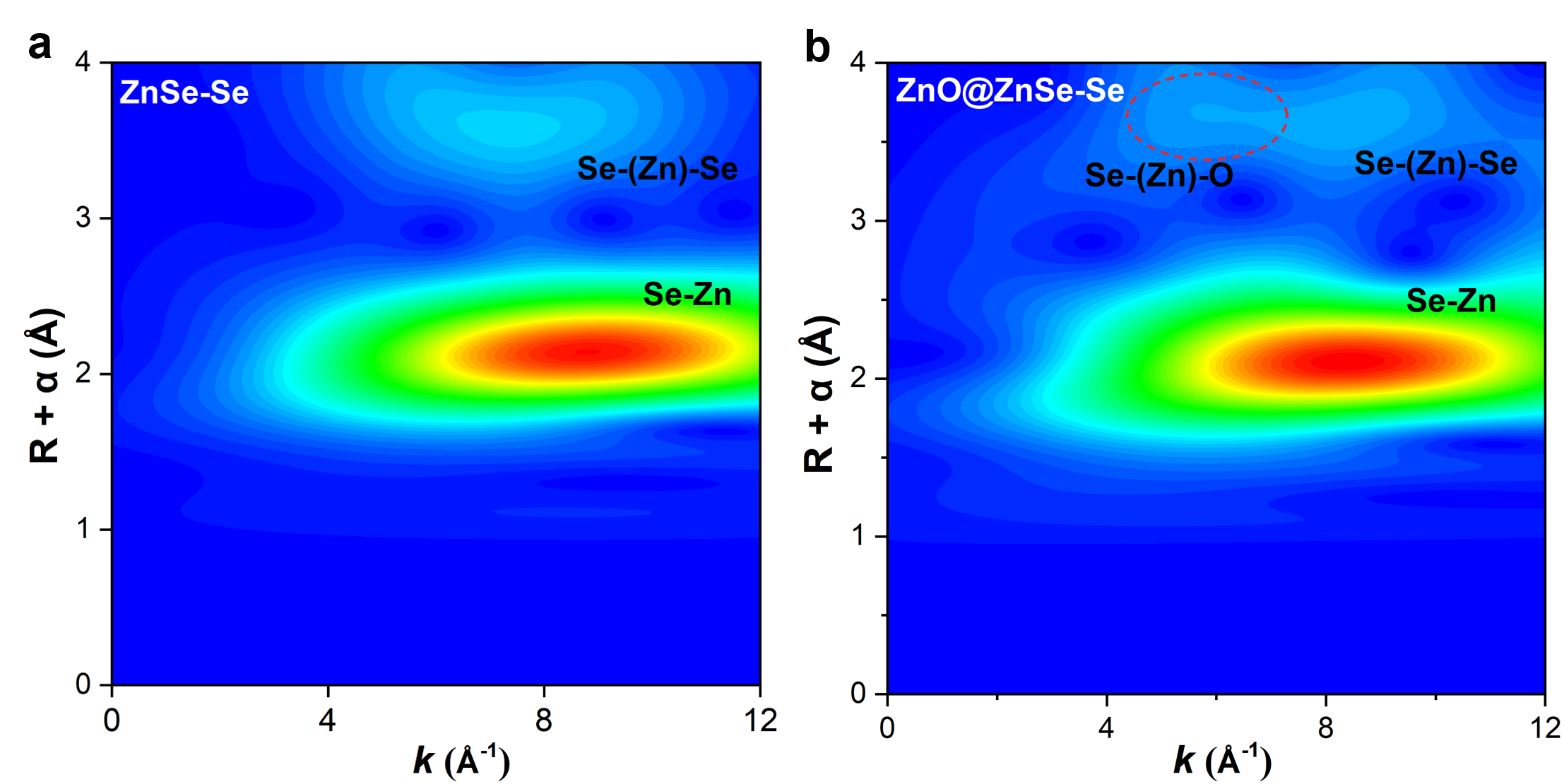
**

**Figure S5**. (a-b) Wavelet transform plot of the Se K-edge EXAFS for ZnSe and ZnO@ZnSe

**
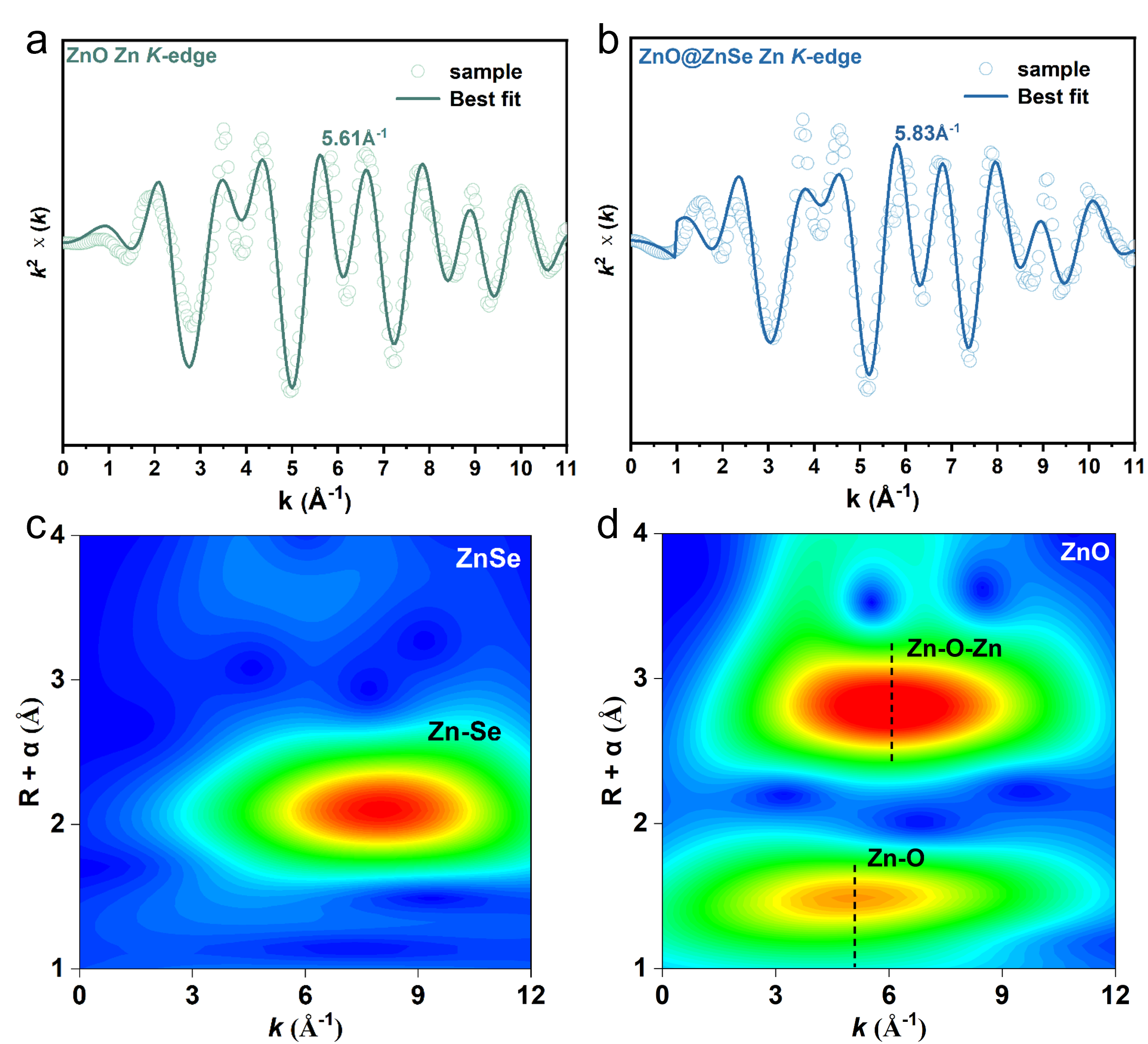
**

**Figure S6**. (a-b) The data in *k*-space for ZnO and ZnO@ZnSe, as well as the best fitting curves, (c-d) the WT-EXAFS graph of ZnSe and ZnO

**
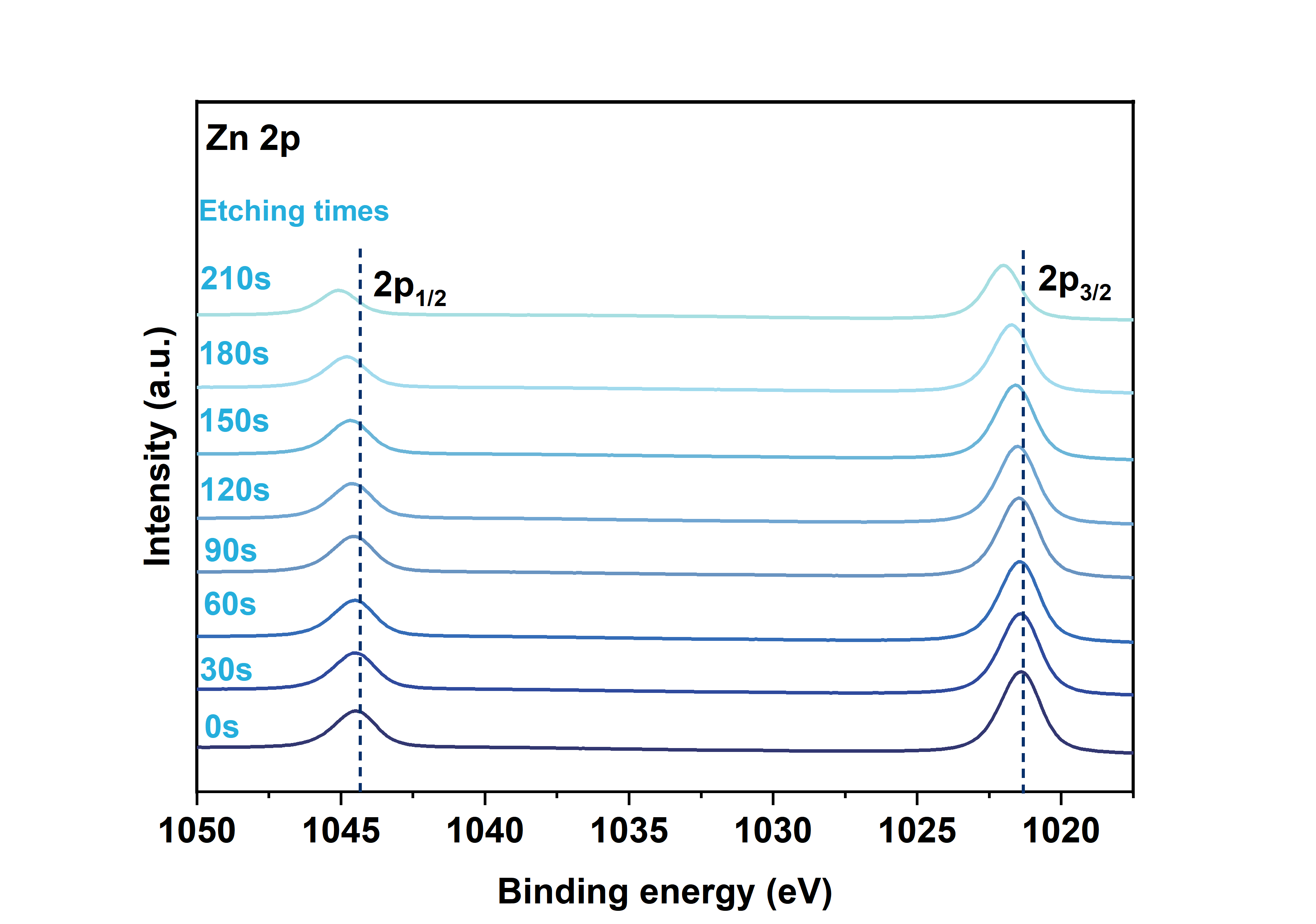
**

**Figure S7**. The fine spectrum of the Zn 2p orbital adopts the etching mode of a single argon ion.

**
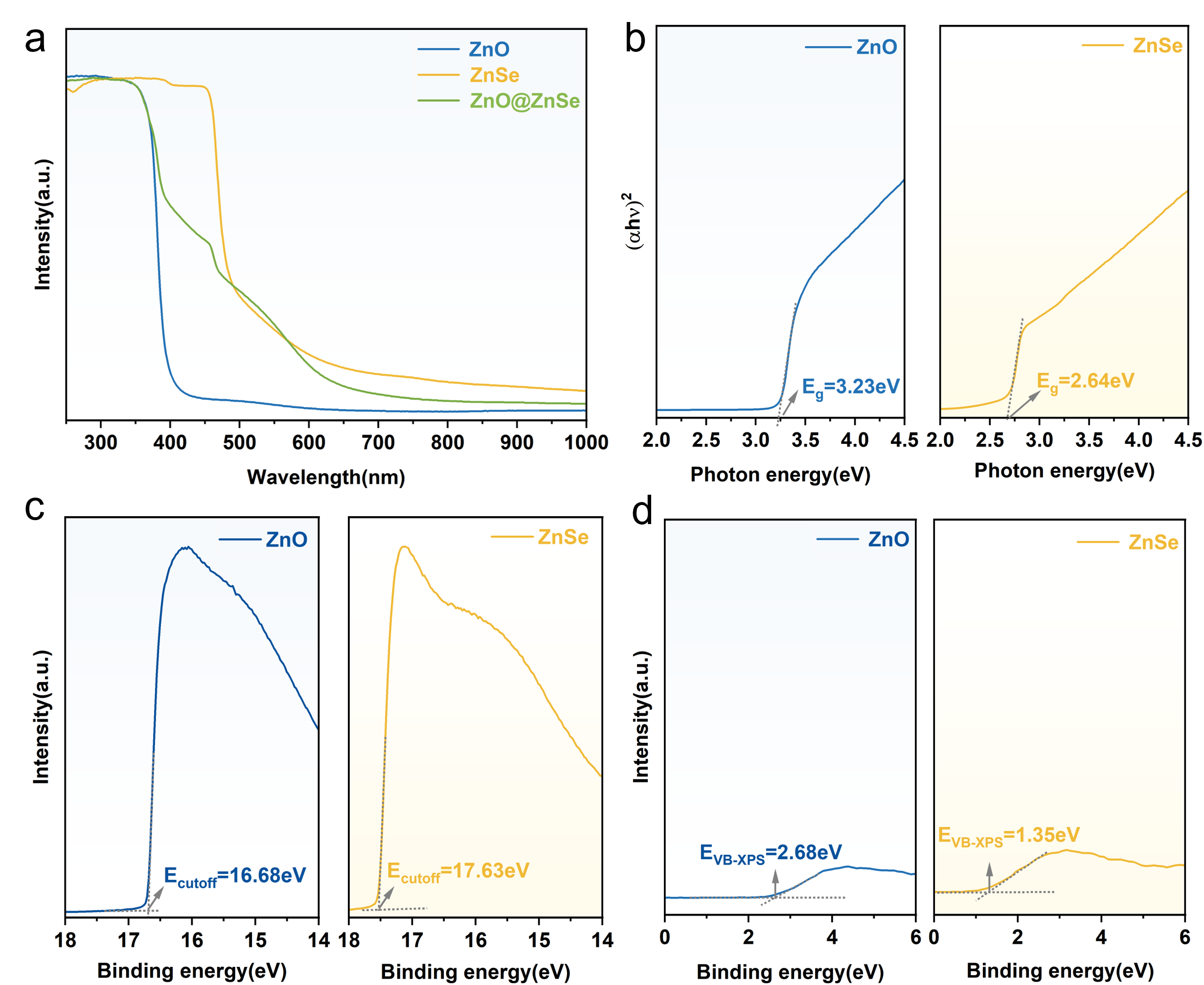
**

**Figure S8**. Analysis of ZnO and ZnSe band structure (a) UV-vis test of ZnO ZnSe and ZnO@ZnSe. (b)E***g*** of ZnO and ZnSe. (c) E*_cutoff_* of ZnO and ZnSe. (d) VB-XPS tests of ZnO and ZnSe

Based on the solid-state UV test results and the Kubelka-Munk formula, (αhν)² = K(*hν* − E*g*), the band gaps of ZnO and ZnSe were calculated to be 3.23 eV and 2.64 eV, respectively. The position of the Fermi level relative to the vacuum level was determined using Ultraviolet Photoelectron Spectroscopy (UPS), calculated according to Equation 1 below, and then converted to the NHE scale, yielding the Fermi levels of ZnO and ZnSe. The valence band (VB) positions of the samples were determined using VB-XPS testing, and the results required correction using the instrument's work function of 0.06 eV vs. NHE. The conduction band (CB) positions of the samples can be calculated using Equation 2 provided below.

W*_function_* = *hv* – E*_cutoff_*

E*_CBM_* = E*_VBM_* - E*g*

**
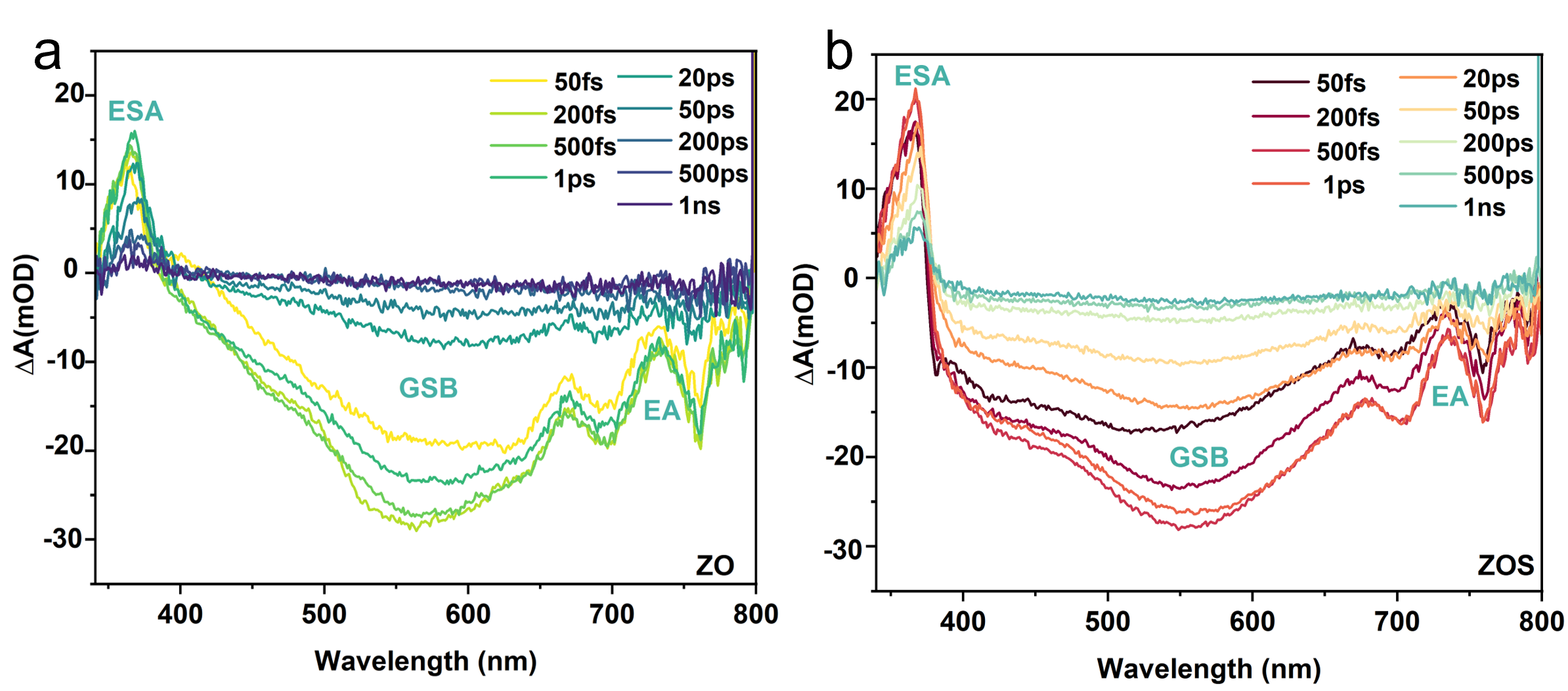
**

**Figure S9**. Two-dimensional ultrafast spectroscopic signals of ZO and ZOS samples acquired at varying relaxation times

**
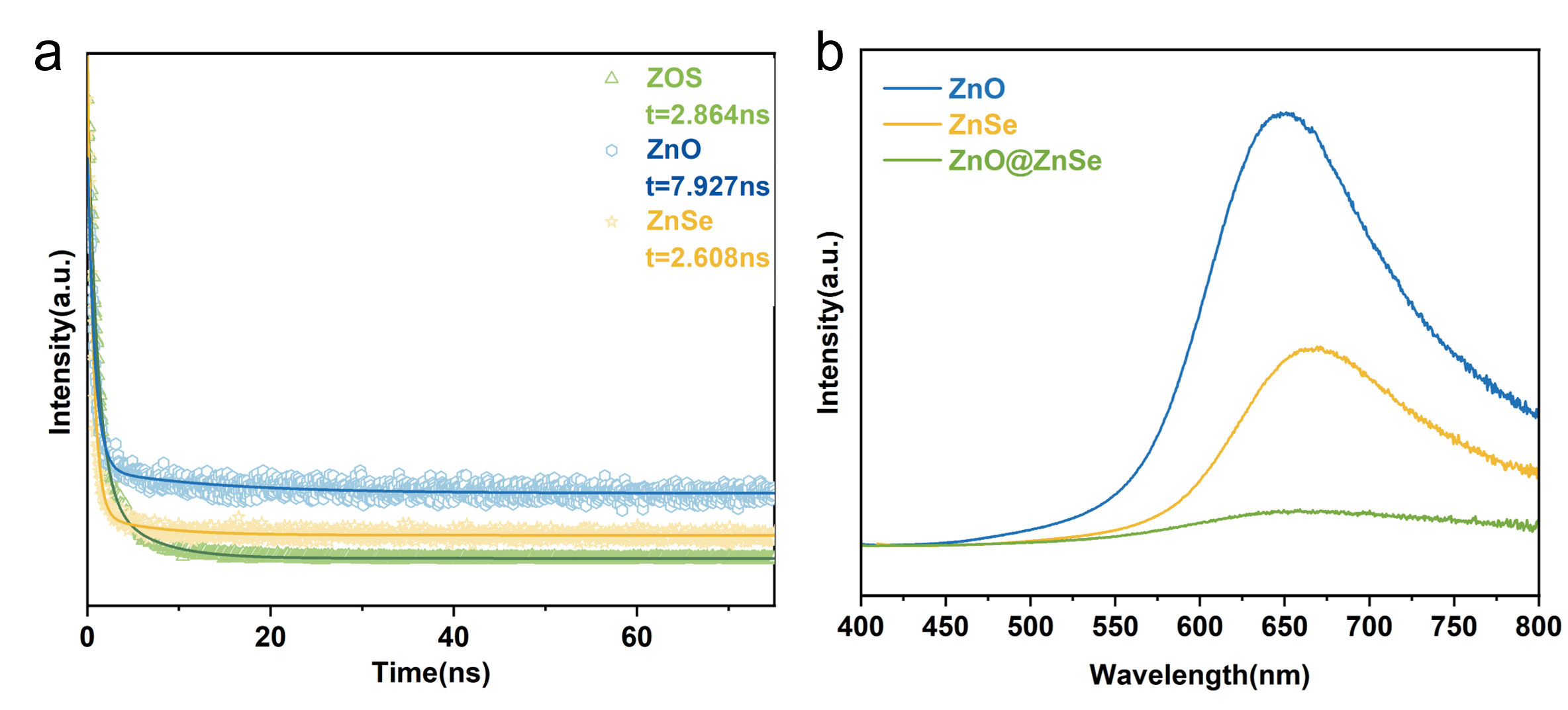
**

**Figure S10**. The TR-PL nad PL test results of ZnO, ZnSe and ZnO@ZnSe

**
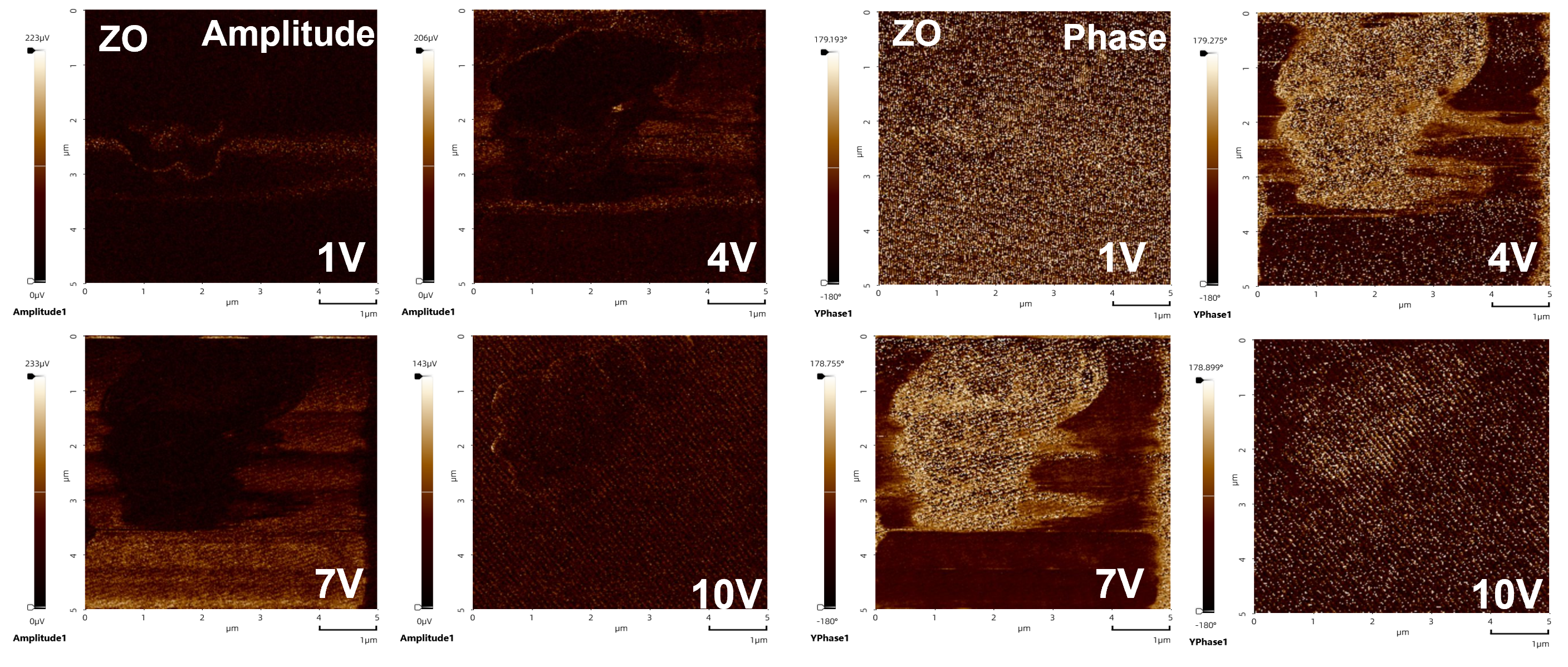
**

**Figure S11**. The PFM test results of ZnO, amplitude and phase diagrams under different bias voltages


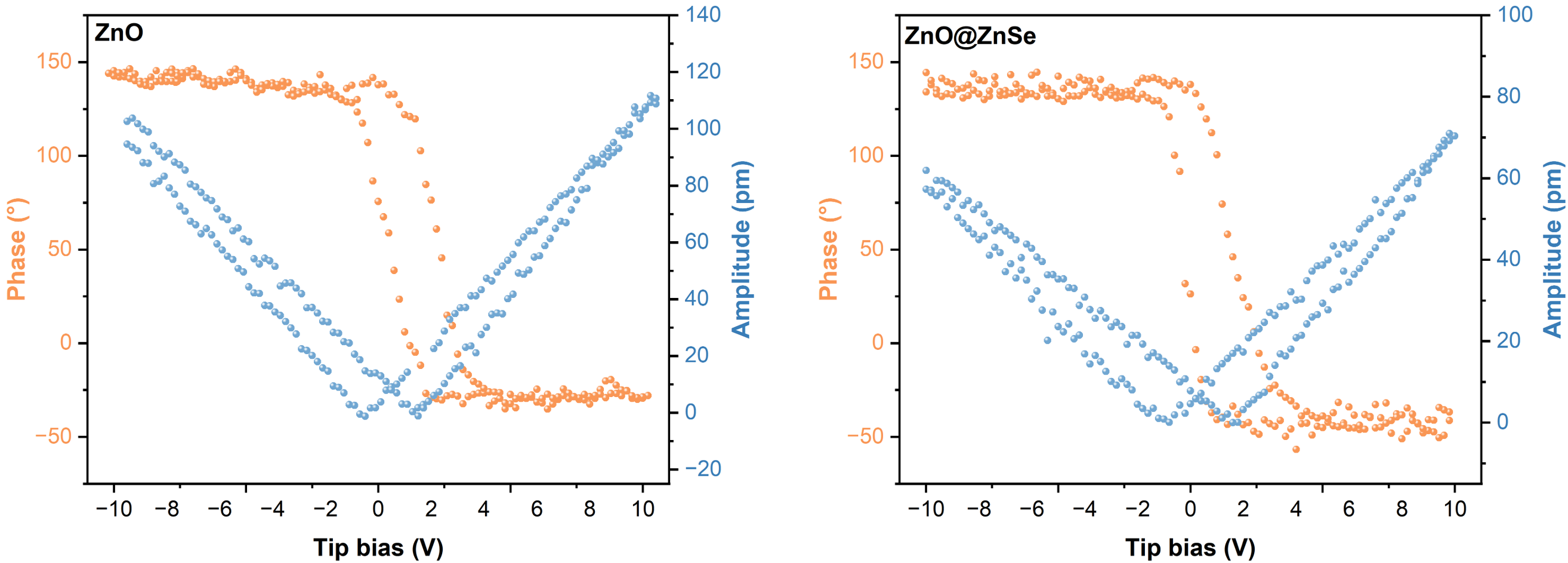


**Figure S12**. The piezoelectric amplitude butterfly curves and phase hysteresis loops of ZnO and ZnO@ZnSe.

**
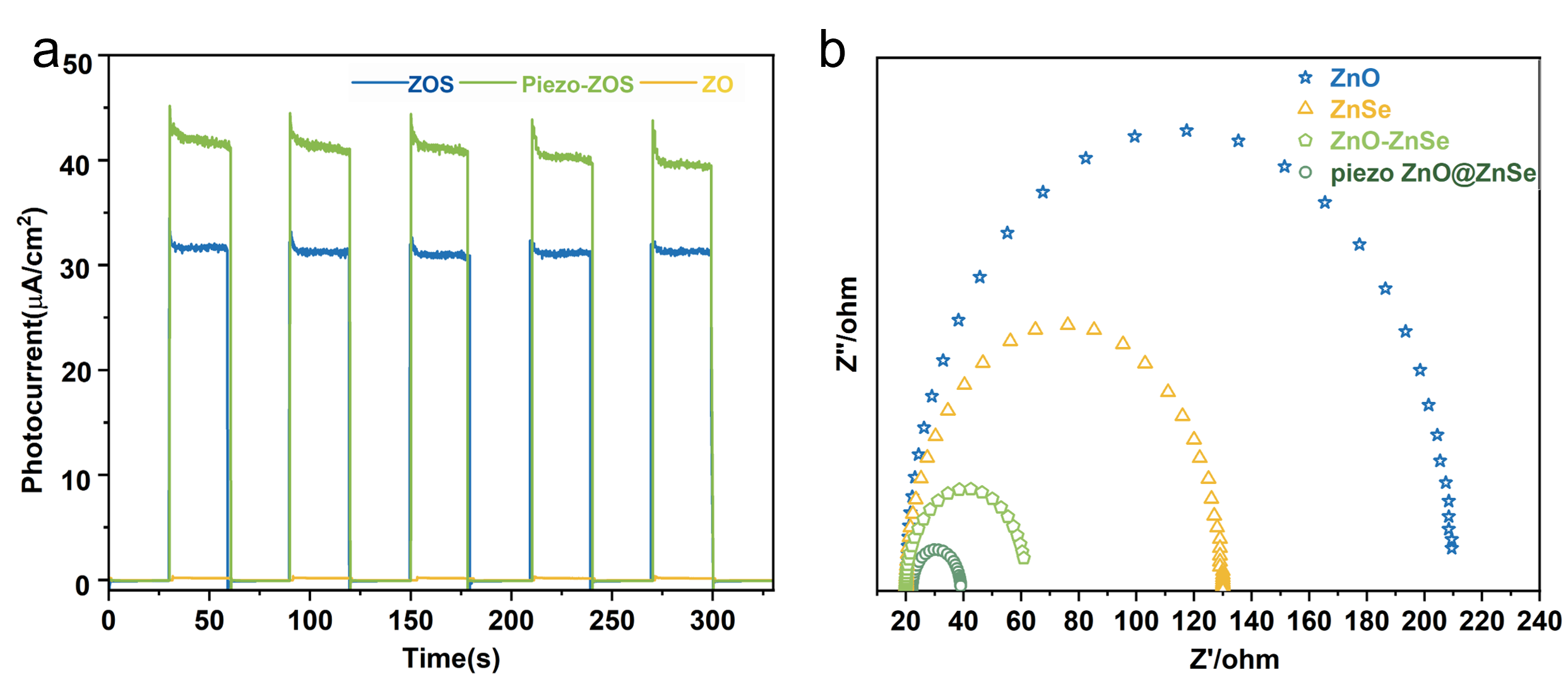
**

**Figure S13**. (a) photocurrent and piezo-photocurrent tests of ZOS, ZO catalysts. (b) Nyquist plot of ZnO ZnSe and ZnO@ZnSe

**
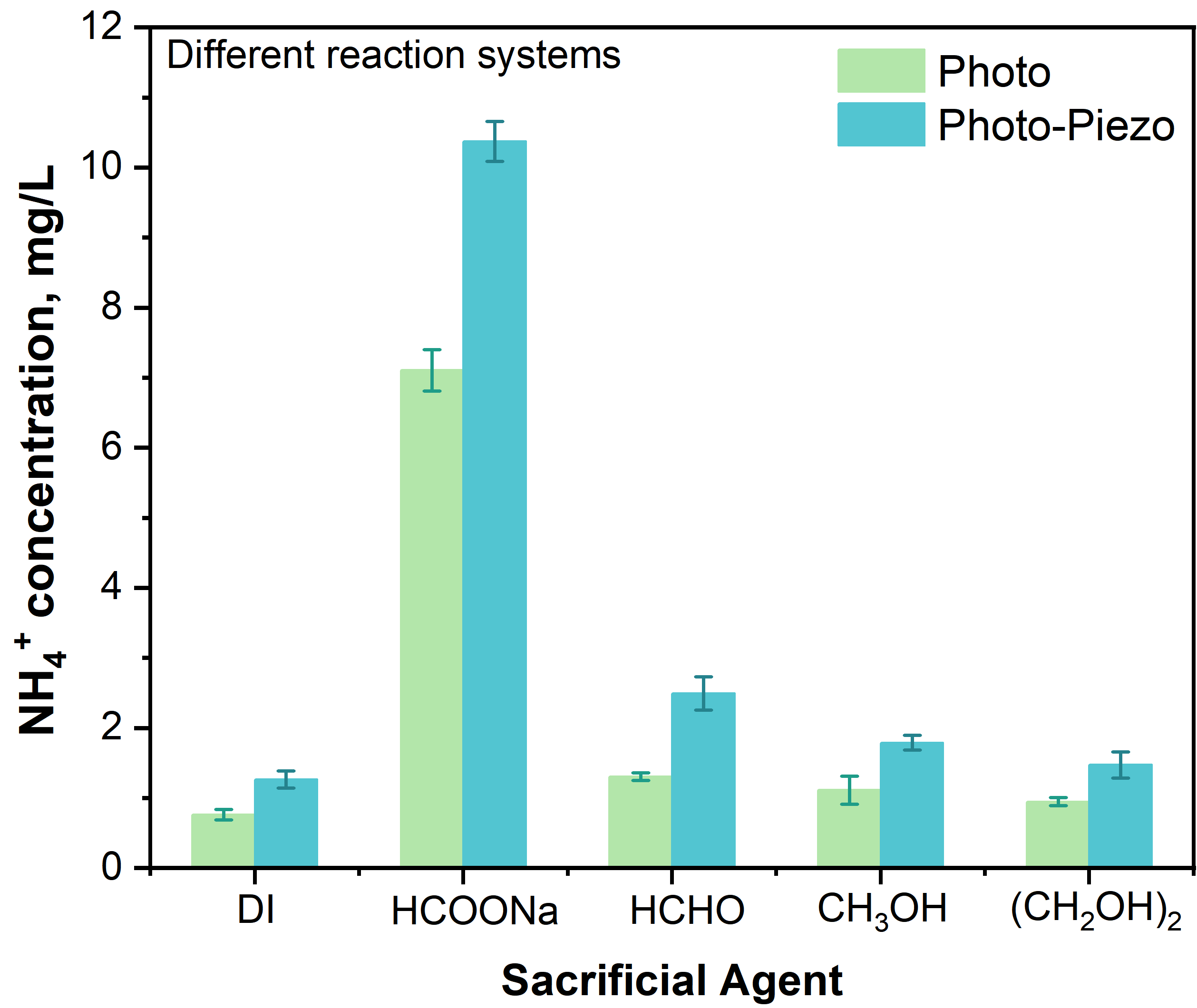
**

**Figure S14**. NH_4_^+^ yield with different sacrificial agents

**
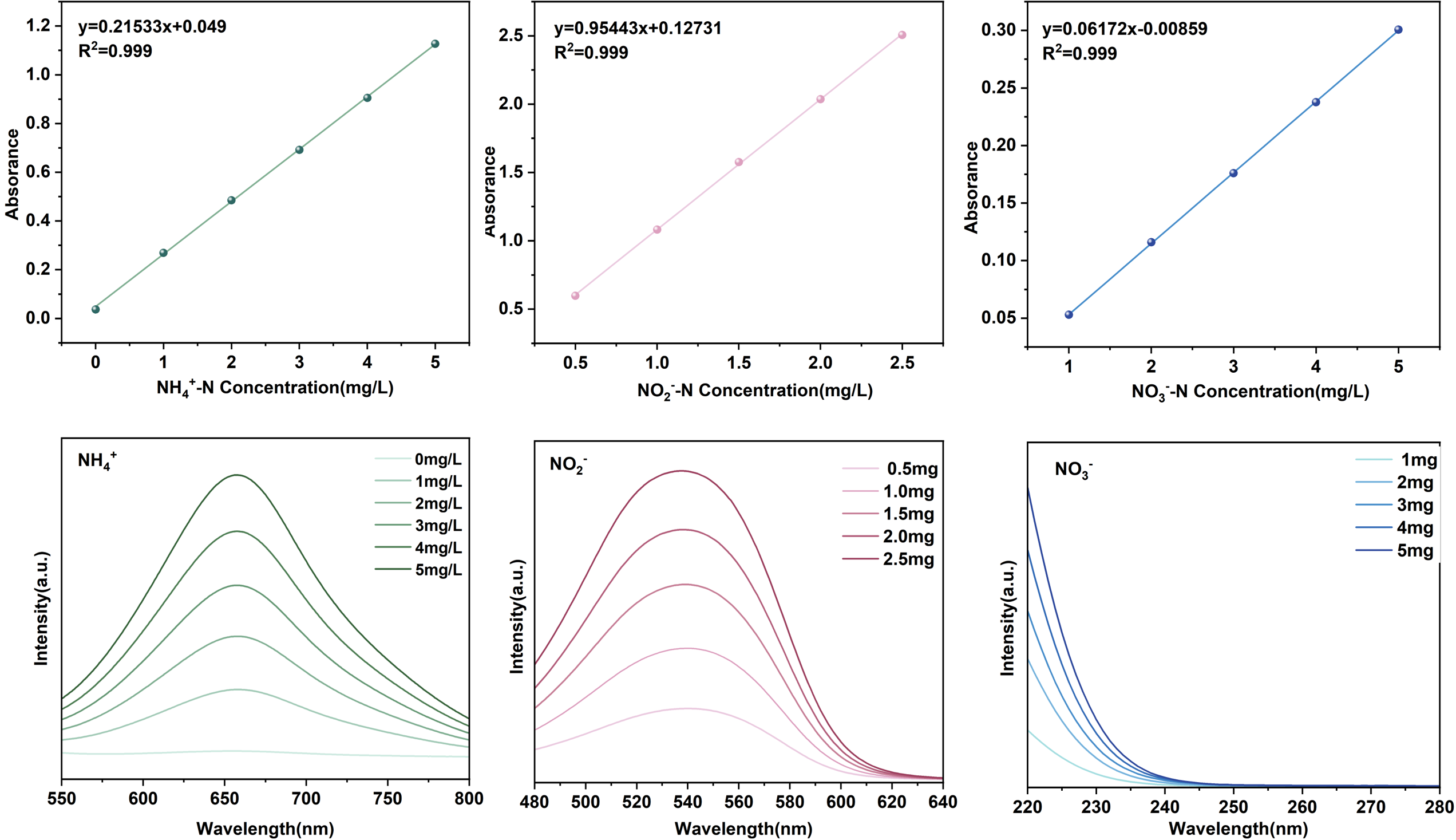
**

**Figure S15**. The calibration curve of the product

**
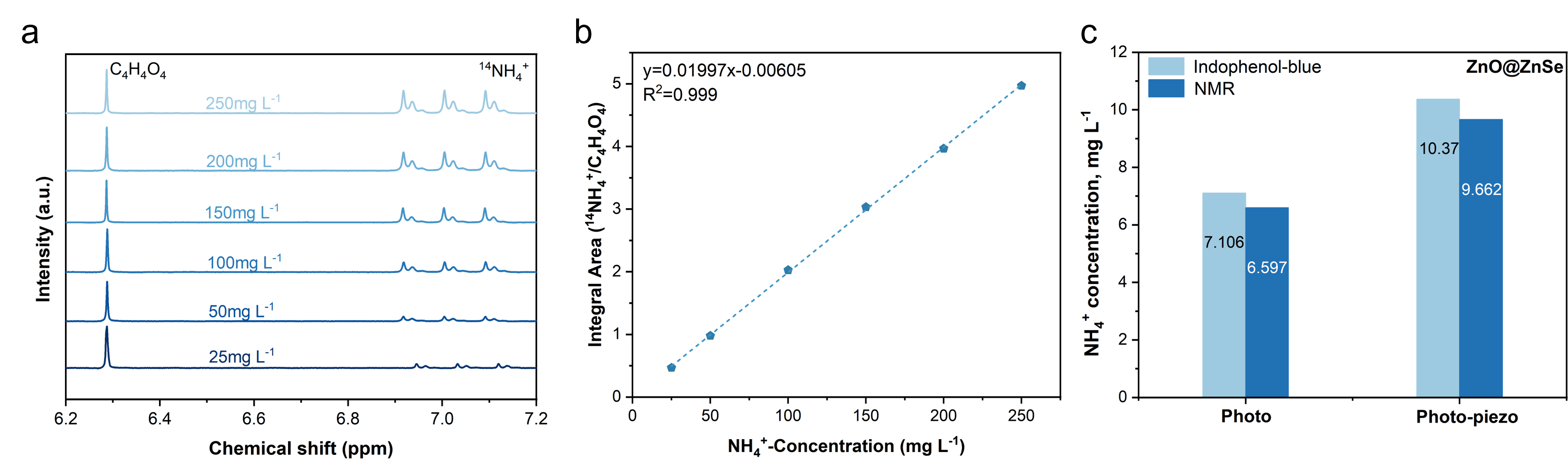
**

**Figure S16.** (a) ^1^H NMR spectra of NH_4_^+^ at different concentrations. (b) Standard curve for NH_4_^+^ concentration determined by the ^1^H NMR method. (c) Performance comparison between the ^1^H NMR method and the indophenol blue method

**
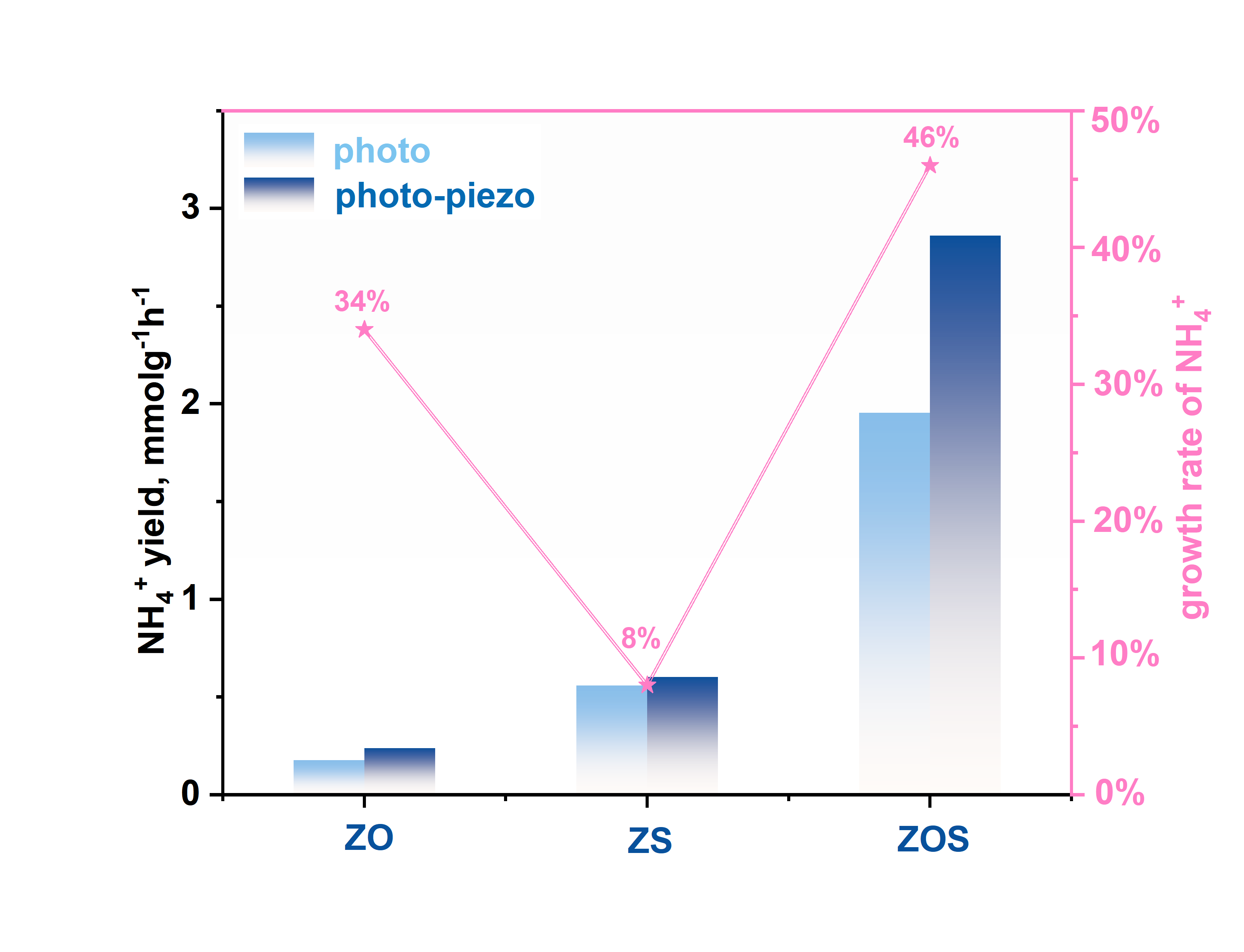
**

**Figure S17**. The yields and growth rates of the ZnO, ZnSe and ZnO@ZnSe catalysts under photocatalysis and piezo-photocatalysis

**
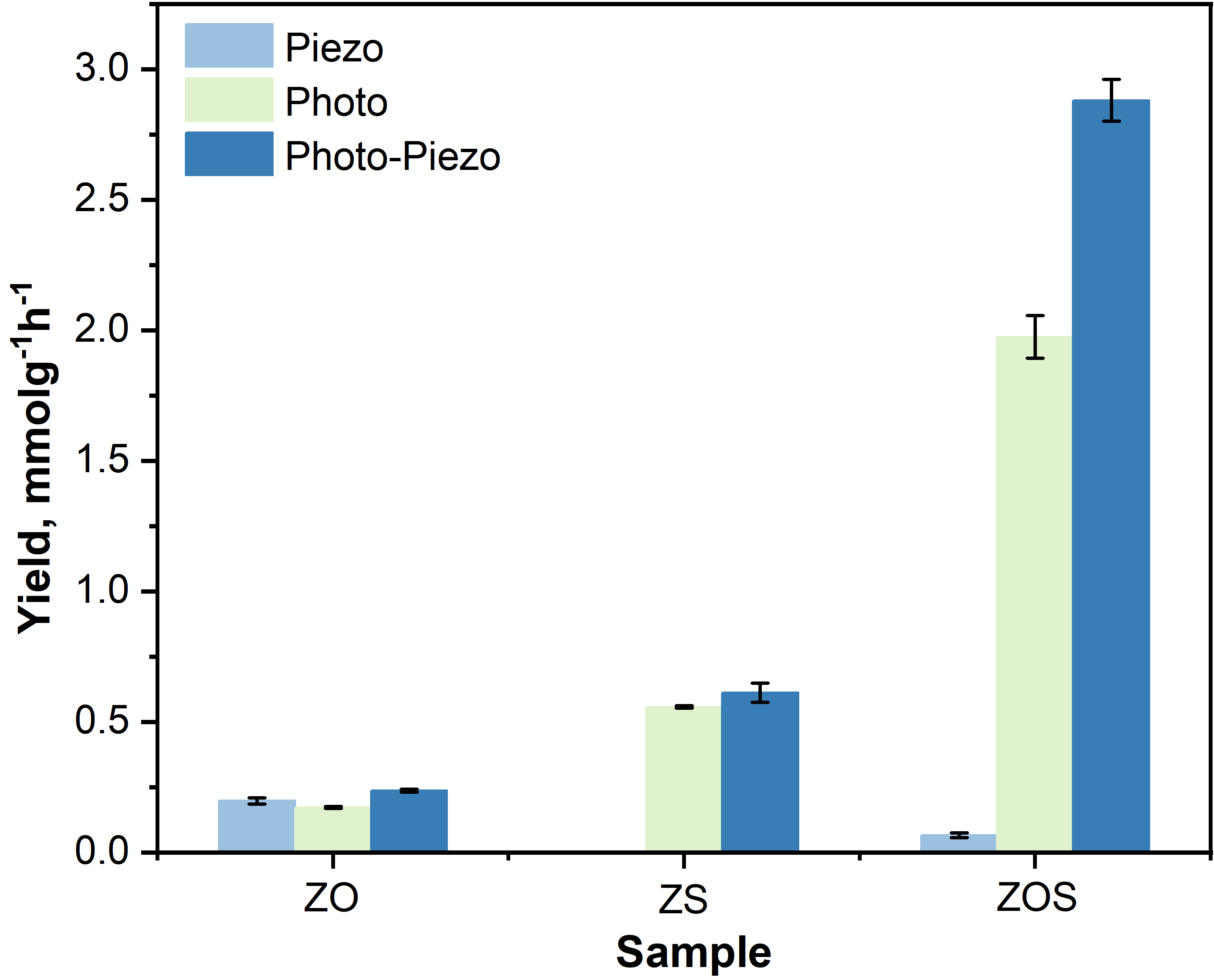
**

**Figure S18.** Performance comparison of ZnO, ZnSe, and ZnO@ZnSe catalysts under piezocatalytic, photocatalytic, and piezo-photocatalytic conditions.

**
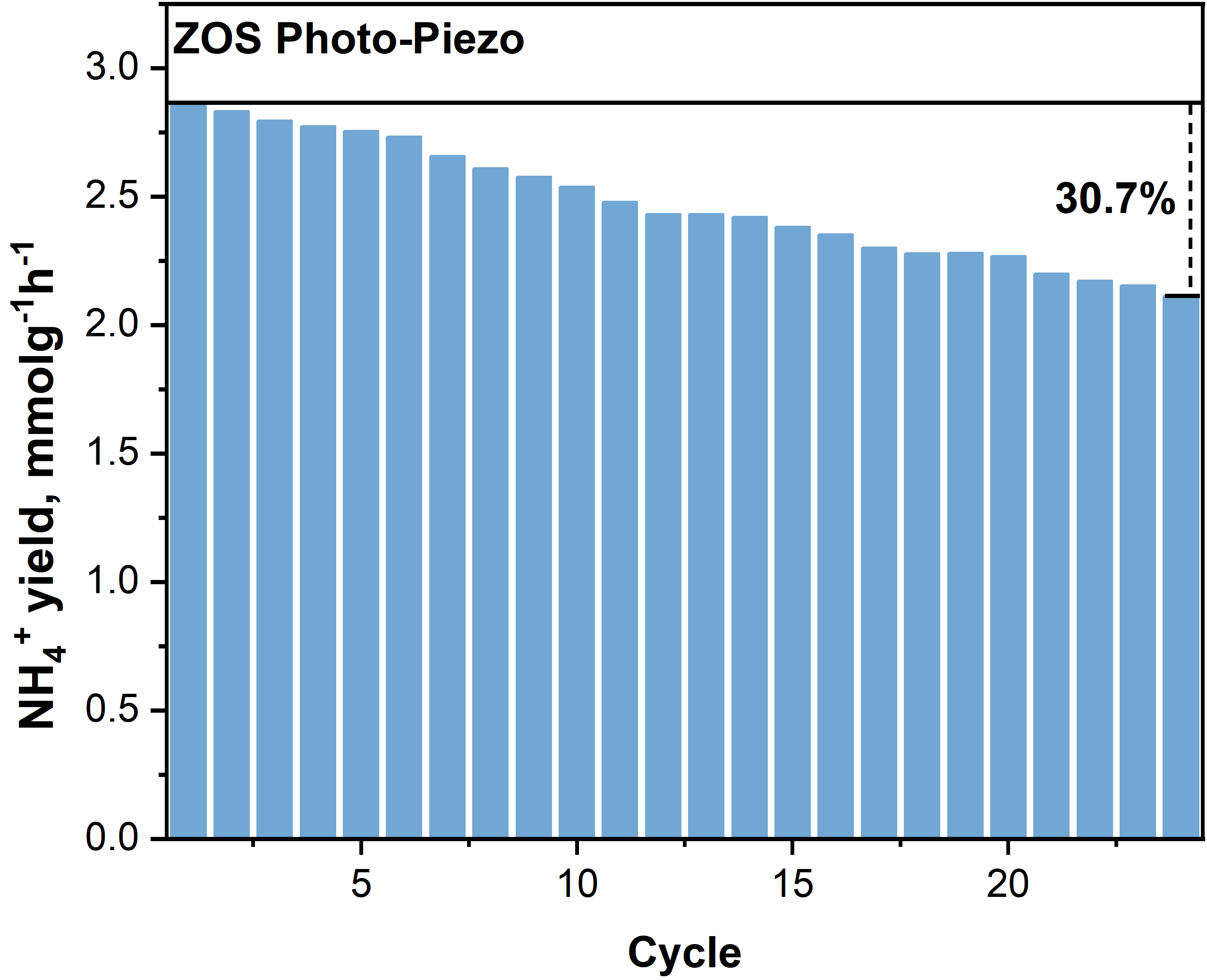
**

**Figure S19.** Long-term stability testing of the ZOS catalyst following 24 cycles.

**
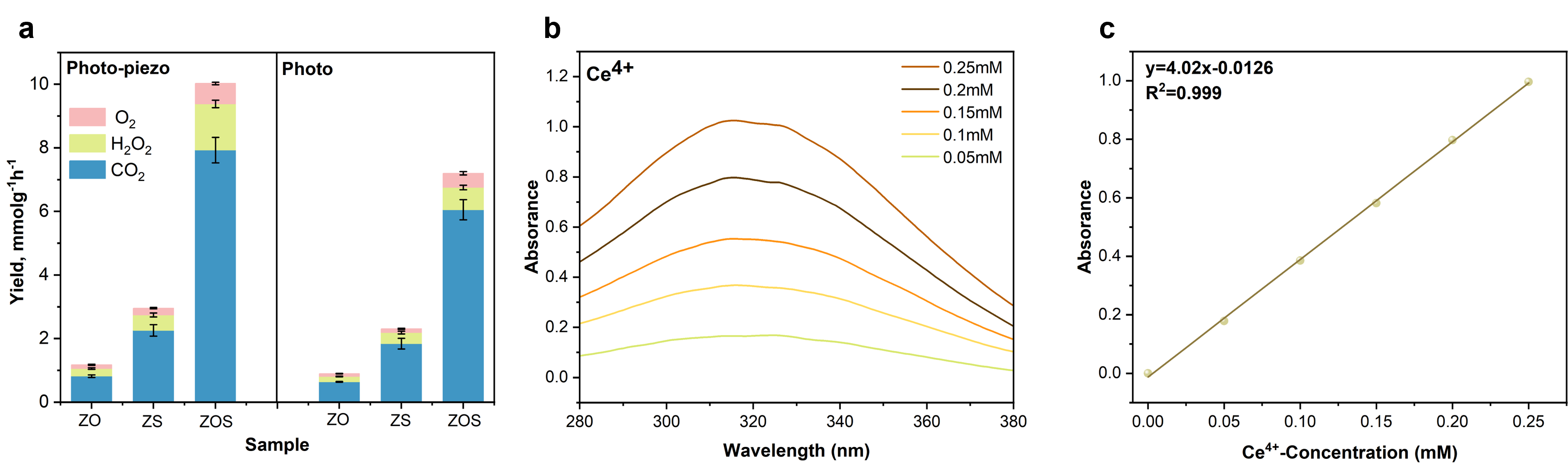
**

**Figure S20.** (a) Stoichiometry of the oxidation products. (b-c) Standard curve of Ce⁴⁺

**
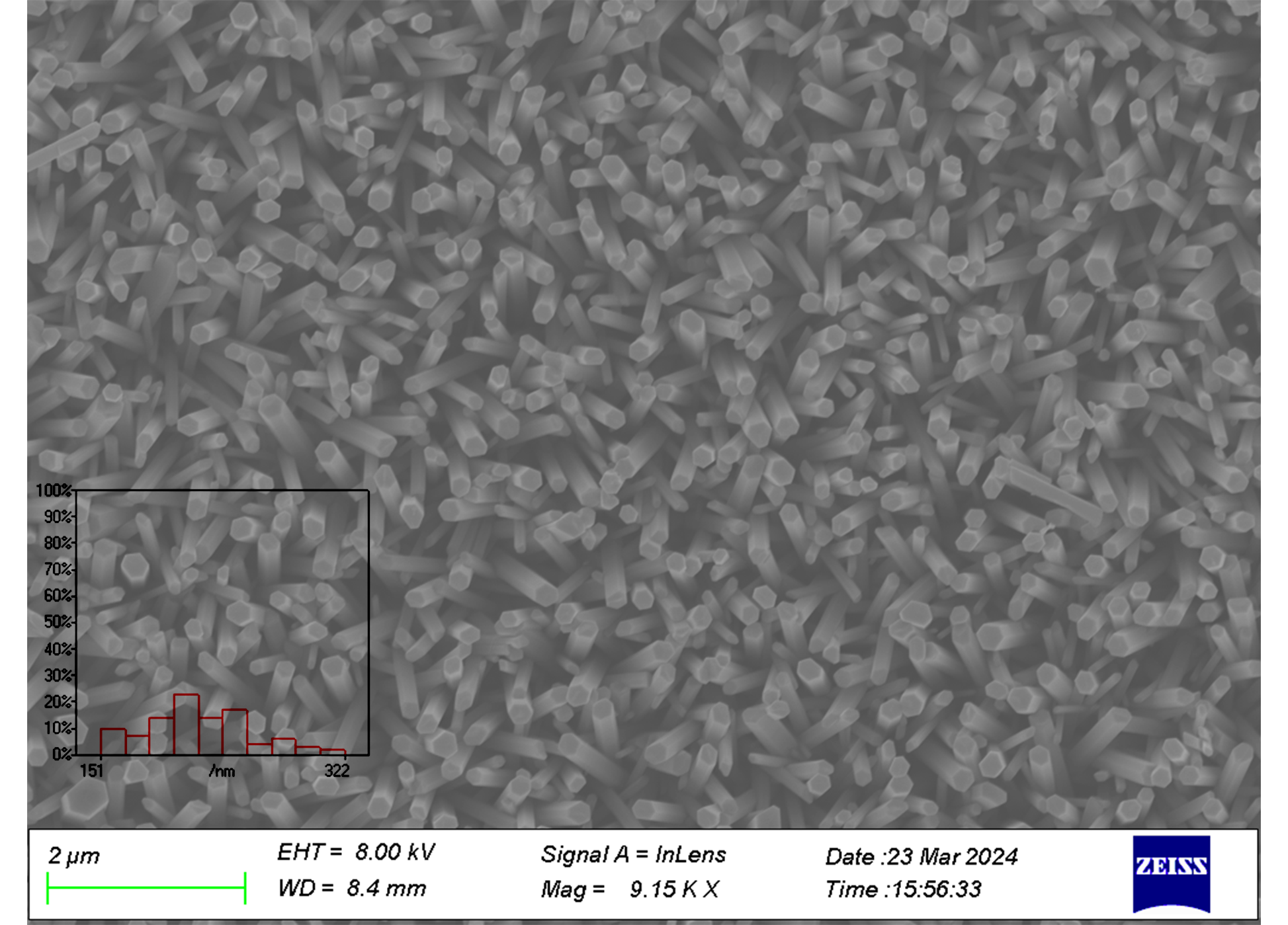
**

**Figure S21**. Statistical analysis of the diameter of ZnO nanorods


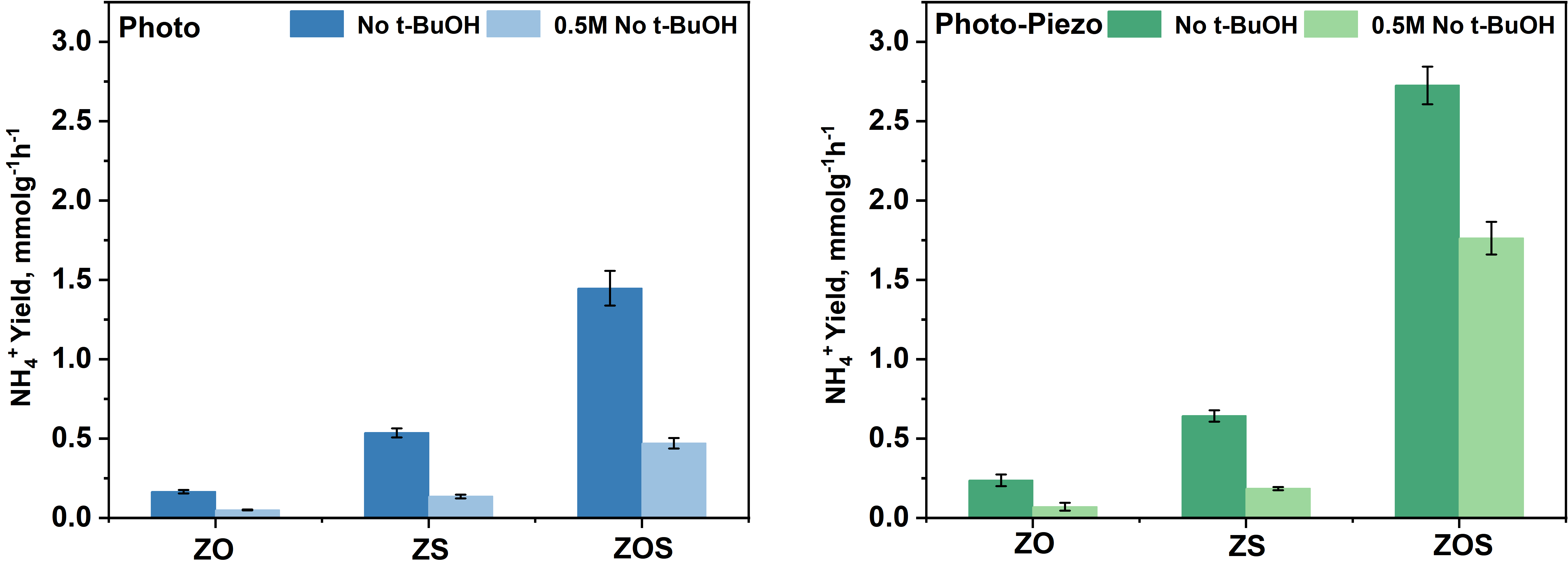


**Figure S22**. Experimental Results of *H Capture

**
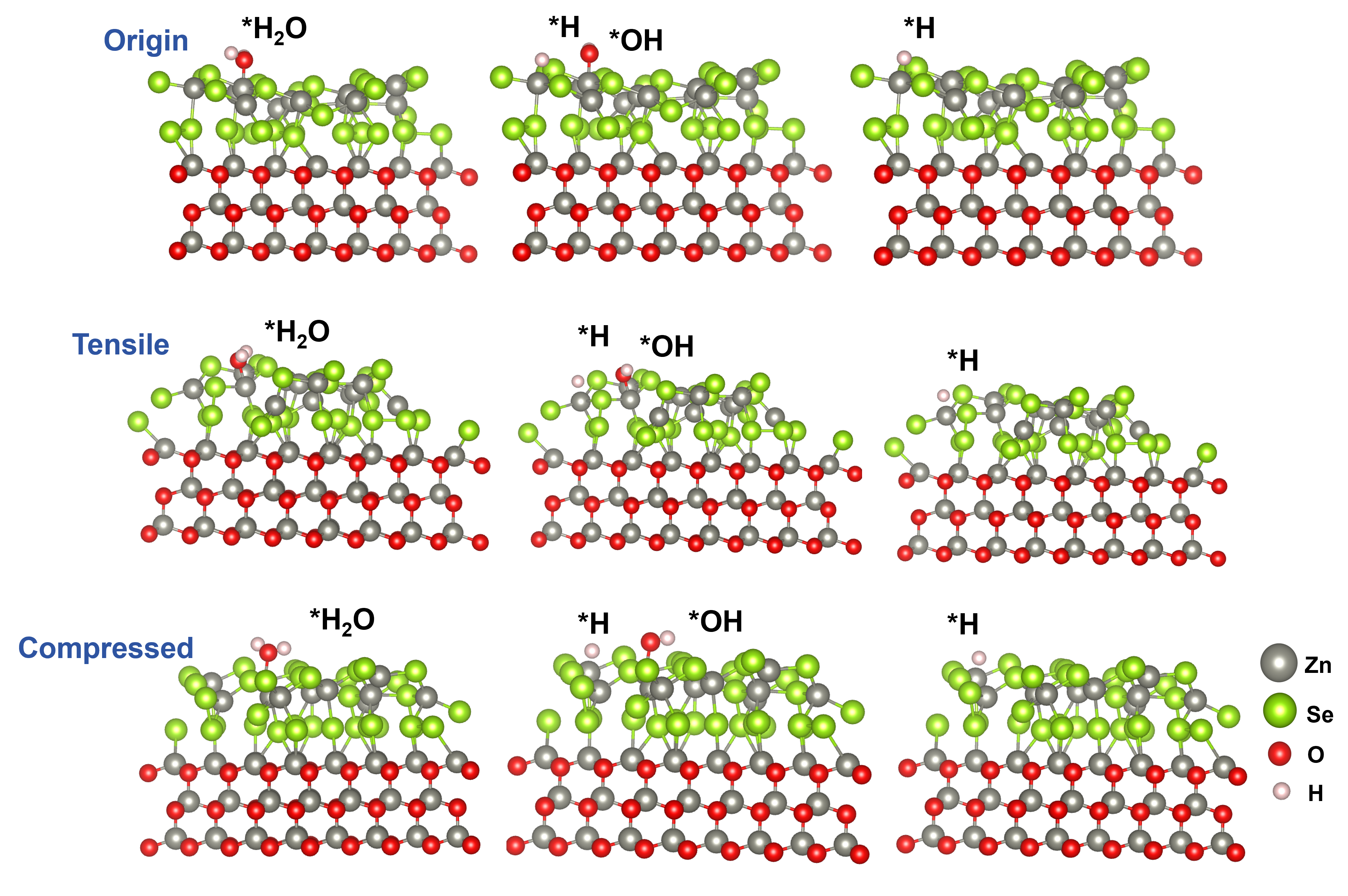
**

**Figure S23.** The configurations of the adsorbed intermediates during hydrolysis under different stress conditions

**
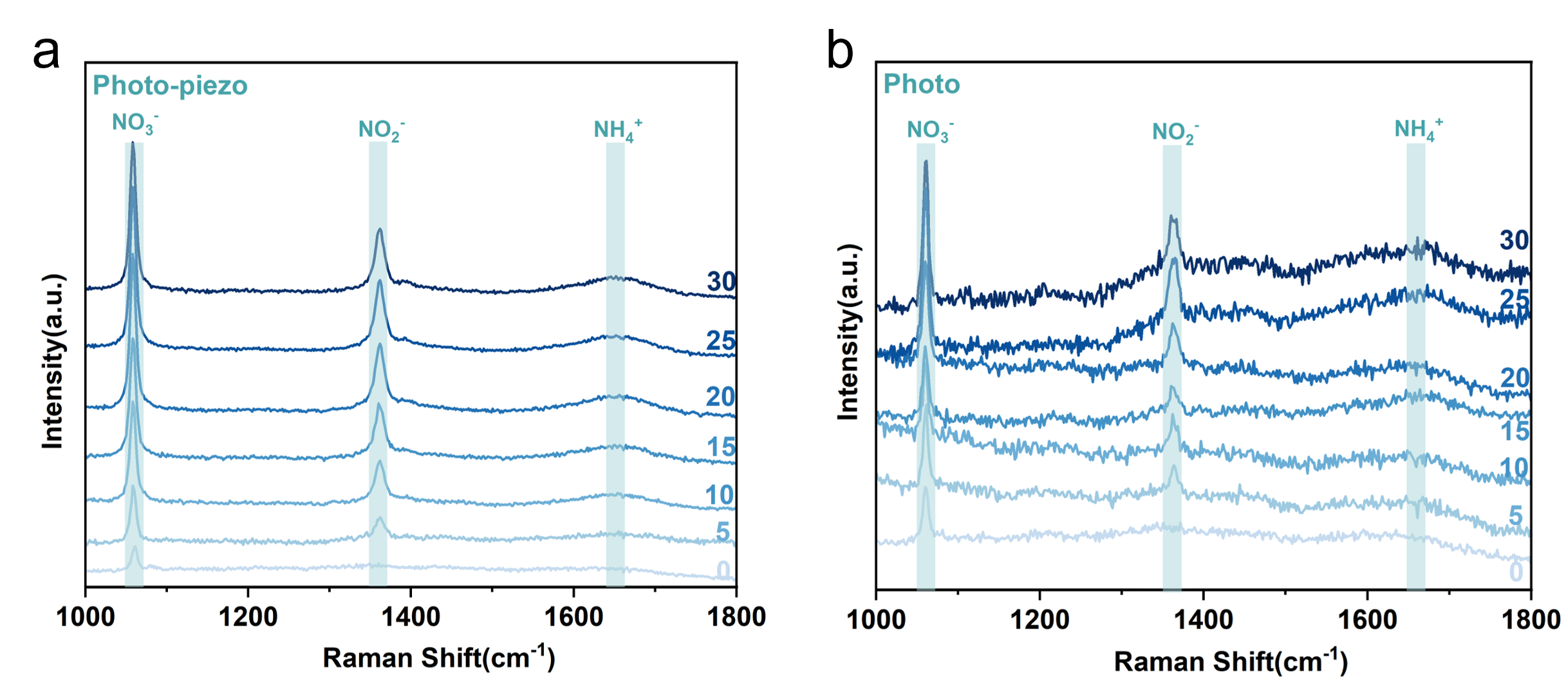
**

**Figure S24**. The in situ Raman test results of the ZnO@ZnSe catalyst under piezo-photocatalysis and photocatalysis conditions

**
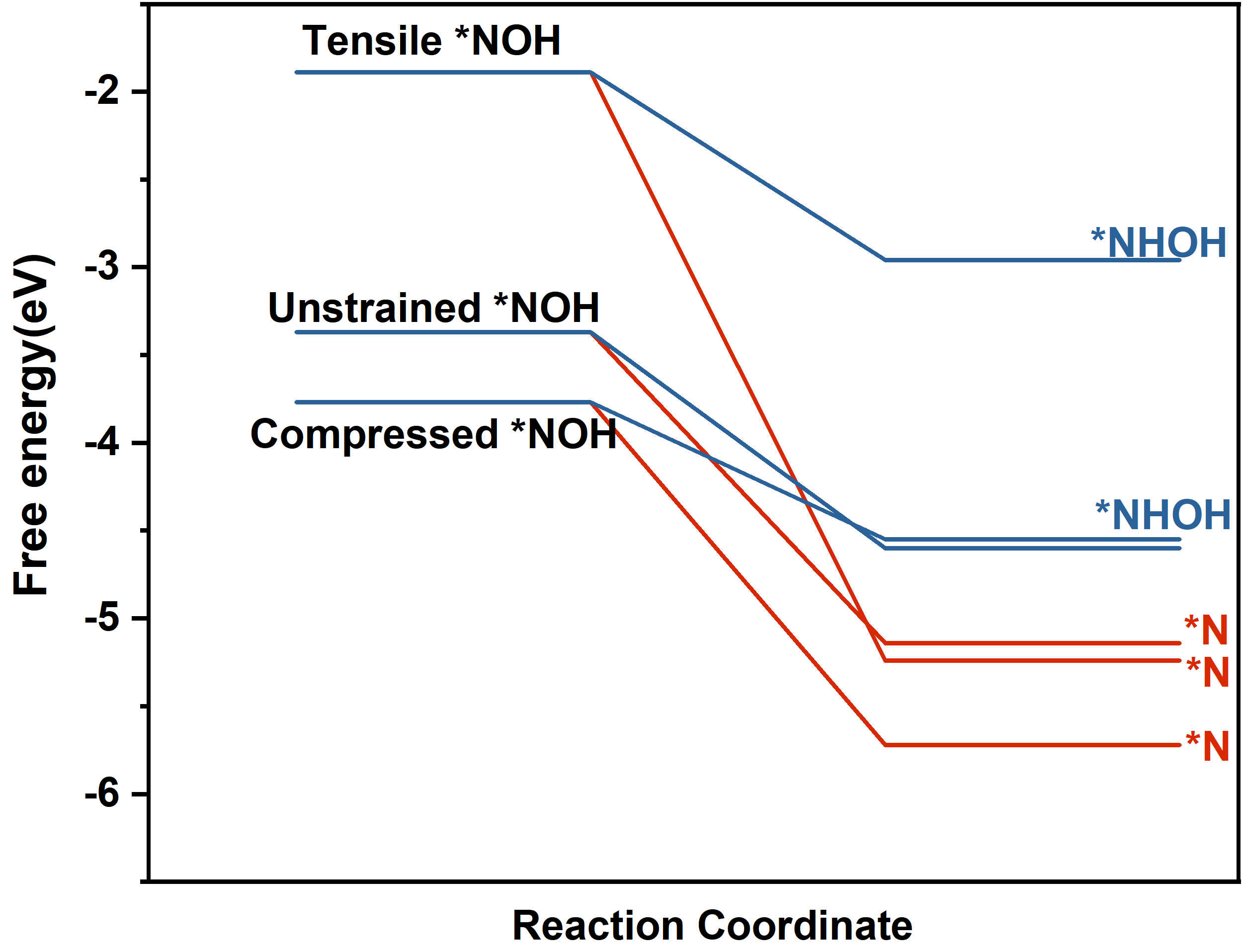
**

**Figure S25.** The Gibbs free energy of the two competing configurations, *N and *NHOH

**
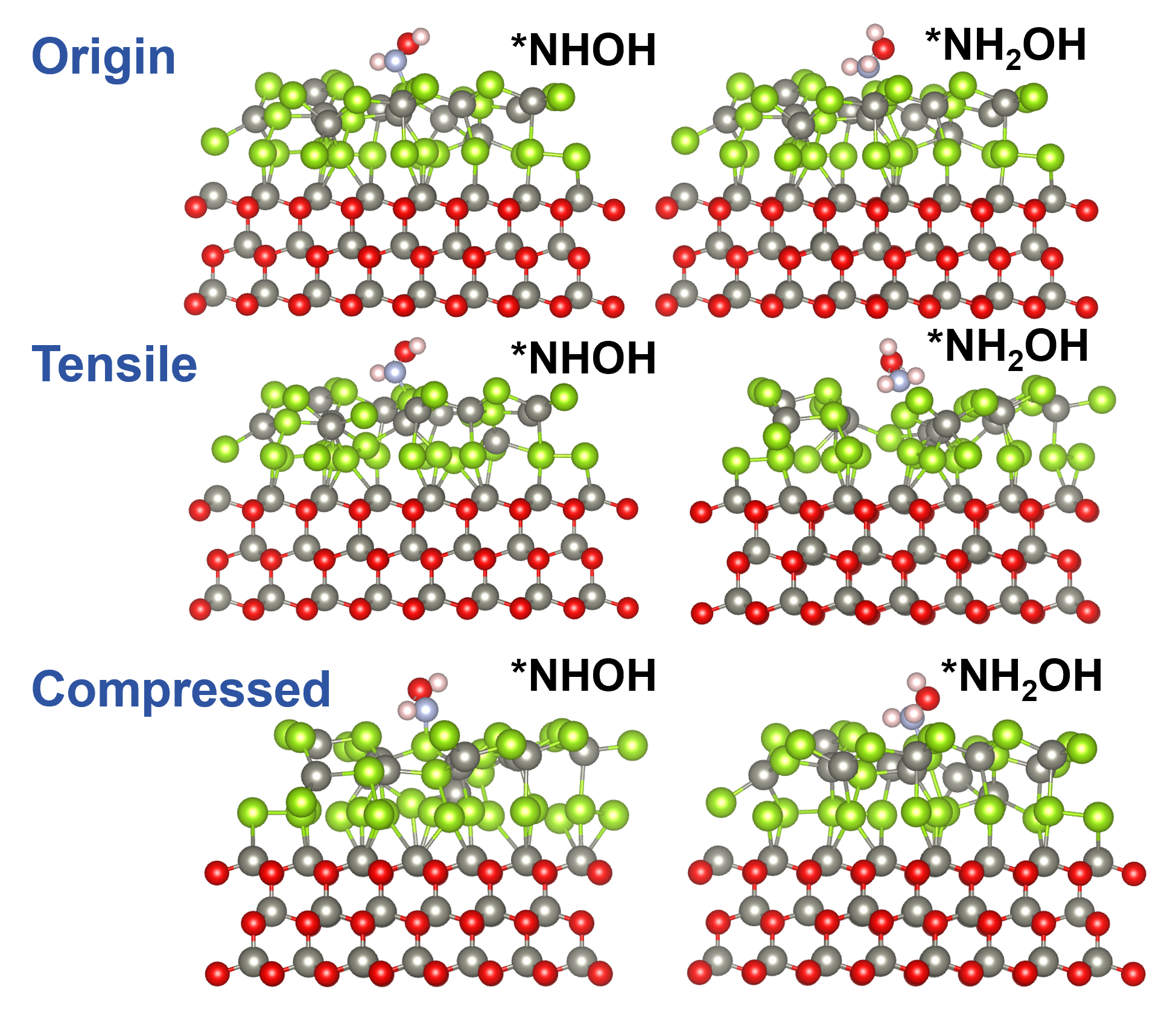
**

**Figure S26**. The adsorption configurations of nitrate reduction intermediates under different stress conditions

**
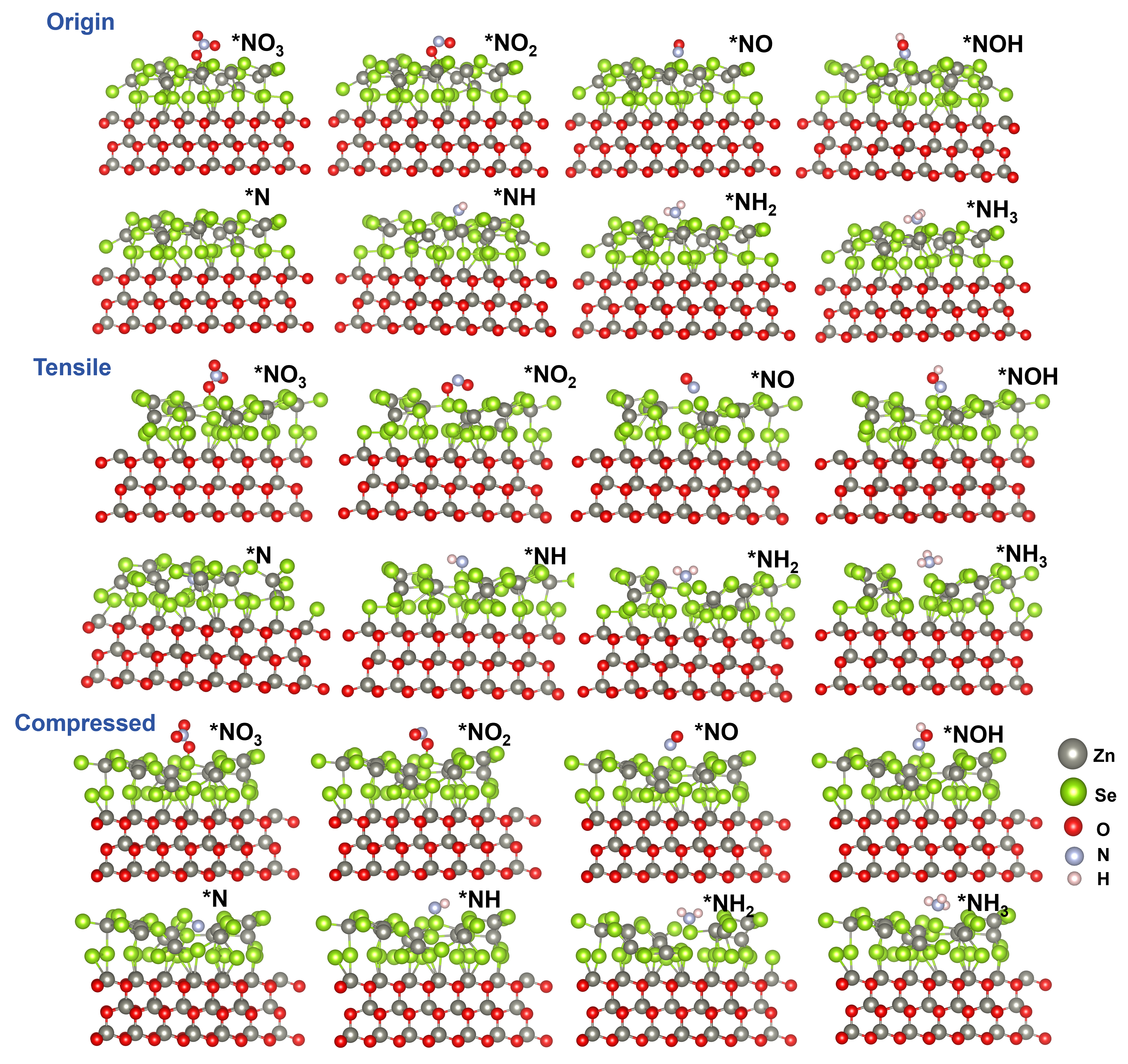
**

**Figure S27.** The adsorption configurations of nitrate reduction intermediates under different strain conditions.

**Supplementary Tables**

Table S1. The specific fitting results of Se K-edge FT-EXAFS

(S_0_^2^=0.91)

| **Sample** | **Shell** | **Bond length**  **(Å)***^a^* | **Coordination**  **Number***^b^* | **σ^2^**  **(Å^2^)***^c^* | **E_0_ shift**  **(eV)***^d^* | **R-factor** |
| --- | --- | --- | --- | --- | --- | --- |
| Se foil | Se-Se | 2.37±0.0058 | 2* | 0.004±0.0007 | 7.05±1.41 | 0.015 |
| SeO_2_ | Se-O | 1.73±0.024 | 1* | 0.006±0.004 | 7.48±3.25 | 0.014 |
| ZnSe | Se-Zn | 2.45±0.048 | 4* | 0.005±0.001 | 2.67±0.64 | 0.011 |
|  | Se-Se | 4.02±0.06 | 12* | 0.017±0.002 |  |  |
| ZnO@ZnSe | Se-Zn | 2.43±0.056 | 3.23±0.26 | 0.006±0.001 | 4.72±0.92 | 0.009 |
|  | Se-O-Se | 3.22±0.09 | 2.18±1.73 | 0.019±0.004 |  |  |
|  | Se-Se | 3.98±0.09 | 11.3±2.95 | 0.017±0.003 |  |  |

*^a^*Bond length, distance between absorber and backscatter atoms; *^b^*Coordination Number; *^c^σ*^2^, Debye-Waller factor to account for both thermal and structural disorders; *^d^E_0_*, inner potential correction; *R* factor indicates the goodness of the fit. *S*_0_^2^ was fixed to 0.91, according to the experimental EXAFS fit of Se foil by fixing coordination number as the known crystallographic value. Fitting range: 3.0 ≤ *k* (Å^-1^) ≤ 11.0 and 1.0 ≤ *R* (Å) ≤ 3.0 (Se foil, SeO_2_); 3.0 ≤ *k* (Å^-1^) ≤ 13.0 and 1.0 ≤ *R* (Å) ≤ 4.1 (ZnSe, ZnO@ZnSe). A reasonable range of EXAFS fitting parameters: 0.700 < *Ѕ*_0_^2^ < 1.000; *CN >* 0; *σ*^2^ > 0 Å^2^; Δ*E*_0_ < 10 eV; *R* factor < 0.02.

**Table S2.** The specific fitting results of Zn K-edge FT-EXAFS

(S_0_^2^=0.87)

| **Sample** | **Shell** | **Bond length**  **(Å)***^a^* | **Coordination**  **Number***^b^* | **σ^2^**  **(Å^2^)***^c^* | **E_0_ shift**  **(eV)***^d^* | **R-factor** |
| --- | --- | --- | --- | --- | --- | --- |
| Zn foil | Zn-Zn | 2.66±0.04 | 6* | 0.009±0.004 | -3.2±2.4 | 0.018 |
|  | Zn-Zn | 2.91±0.01 | 6* | 0.017±0.014 |  |  |
| ZnO | Zn-O | 1.97±0.01 | 4* | 0.004±0.001 | -2.0±1.2 | 0.007 |
|  | Zn-Zn | 3.23±0.01 | 12* | 0.009±0.001 |  |  |
| ZnSe | Zn-Se | 2.44±0.01 | 4* | 0.005±0.001 | 1.6±1.3 | 0.007 |
| ZnO@ZnSe | Zn-O | 2.01±0.01 | 4.3±0.9 | 0.002±0.000 | 3.8±1.0 | 0.020 |
|  | Zn-Zn | 3.27±0.01 | 10.5±2.2 | 0.017±0.003 |  |  |

*^a^*Bond length, distance between absorber and backscatter atoms; *^b^*Coordination Number; *^c^σ*^2^, Debye-Waller factor to account for both thermal and structural disorders; *^d^E_0_*, inner potential correction; *R* factor indicates the goodness of the fit. *S*_0_^2^ was fixed to 0.87, according to the experimental EXAFS fit of Zn foil by fixing coordination number as the known crystallographic value. Fitting range: 3.0 ≤ *k* (Å^-1^) ≤ 12.0 and 1.0 ≤ *R* (Å) ≤ 3.0 (Zn foil, ZnSe); 3.0 ≤ *k* (Å^-1^) ≤ 13.0 and 1.0 ≤ *R* (Å) ≤ 3.5 (ZnO, ZnO@ZnSe). A reasonable range of EXAFS fitting parameters: 0.700 < *Ѕ*_0_^2^ < 1.000; *CN >* 0; *σ*^2^ > 0 Å^2^; Δ*E*_0_ < 10 eV; *R* factor < 0.02.

**Table S3**. The lifetime fitting of ZO, ZOS catalysts at 365nm

| **Sample** | **A_1_** | ***τ*_1(_ps)**  **(Rel. %)** | **A_2_** | ***τ*_2_(ps)**  **(Rel. %)** | **A_3_** | ***τ*_3_(ps) (Rel. %)** |
| --- | --- | --- | --- | --- | --- | --- |
| ZnO | 76.7 | 8.438(2.3%) | 61.3 | 441.292(97.6%) | - | - |
| ZnO@ZnSe | 41.8 | 7.159(2.9%) | 44.2 | 195.07(83.8%) | 28.8 | 47.16(13.2%) |

**Table S4**. The numerical distribution and probability of the diameter of ZnO nanorods

| **Distr./nm** | **Mean/nm** | **Amount** | **Freq.** |
| --- | --- | --- | --- |
| 151-168.1 | 159.55 | 10 | 10% |
| 168.1-185.2 | 176.65 | 7 | 7% |
| 185.2-202.3 | 193.75 | 14 | 14% |
| 202.3-219.4 | 210.85 | 23 | 23% |
| 219.4-236.5 | 227.95 | 14 | 14% |
| 236.5-253.6 | 245.05 | 17 | 17% |
| 253.6-270.7 | 262.15 | 4 | 4% |
| 270.7-287.8 | 279.25 | 6 | 6% |
| 287.8-304.9 | 296.35 | 3 | 3% |
| 304.9-322 | 313.45 | 2 | 2% |

**Table S5**. Performance comparison of ZnO@ZnSe catalysts with other catalysts in photoelectric and piezo-photocatalytic ammonia production.

| **Catalyst** | **Scavengers** | **Optimal ammonia synthesis rate** | **Excitation source** | **Reference** |
| --- | --- | --- | --- | --- |
| ZnO@ZnSe | HOOCNa | 2.88 mmol g⁻¹ h⁻¹ | 300W xenon lamp illumination; 480W ultrasonic frequency. | This work |
| PbTiO_3_  (N_2_ reduction) | No | 0.1089 mmol g^-1^ h^-1^ | 300 W xenon lamp; 40 kHz, 200 W ultrasound. | [5] |
| BaTiO_3_ (OVs)  (N_2_ reduction) | 0.1 M Na_2_S/0.1 M Na_2_SO_3_ | 0.1067 mmol g-1 h^-1^ | simulated solar light; ultrasound (40 kHz, 200 W) | [6] |
| Ru g-C_3_N_4_ | HCOOH | 0.154 mmol g^-1^ h^-1^ | Under simulated solar light at an intensity of 600 mW/cm^2^ | [7] |
| carbon/bismuth/bismuth oxide | No | 0.378 mmol g^-1^ h^-1^ | Under simulated solar light at an intensity of 600 mW/cm^2^ | [8] |
| SACu/TN/CIS SHPs | HCOOH | 0.256 mmol g^-1^ h^-1^ | 250 W xenon lamp | [9] |
| Ag_2_O/P25 | HCOOH | 0.45 mmol g^-1^ h^-1^ | a 300 W high-pressure mercury lamp with a main wavelength of approximately 365 nm | [10] |
| AgCu-CN | ethylene glycol (EG) | 0.6305 mmol g^-1^ h^-1^ | A 300 W xenon lamp equipped with a 420 nm cutoff filter was used as the light source | [11] |
| TiO_2_ P25 | H_2_C_2_O_4_ | 0.69 mmol g^-1^ h^-1^ | illumination from a 400 W UV lamp | [12] |
| CuO_x_-TiO_2_ | HCOOH | 1.639 mmol g^-1^ h^-1^ | illumination from a 300 W xenon lamp | [13] |
| Cu-NM-1.5 | ethylene glycol (EG) | 1.929 mmol g^−1^ h^−1^ | - | [14] |
| CuS_1_N_4_ | ethylene glycol (EG) | 1.979 mmol g^−1^ h^−1^ | A 300 W xenon lamp (320-780 nm) was used as the light source | [15] |
| NH_2_-MIL-125 | No | 2.454 mmol g^−1^ h^−1^ | A 300 W xenon lamp equipped with a 420 nm long-pass  filter | [16] |

**References**

[1] Y. Zhang, S. Wang, Y. Zhao, Y. Ding, Z. Zhang, T. Jiang, Z. L. Wang, L. Li, *Mater. Today Nano* **2022**, 18, 100177.

[2] J. Xu, C.-Y. Luan, Y.-B. Tang, X. Chen, J. A. Zapien, W.-J. Zhang, H.-L. Kwong, X.-M. Meng, S.-T. Lee, C.-S. Lee, *ACS Nano* **2010**, 4, 6064.

[3] D. Li, S. Hussain, Y. Wang, C. Huang, P. Li, M. Wang, T. He, *Appl. Catal. B* **2021**, 286, 119887.

[4] G. Wang, Z. Chen, T. Wang, D. Wang, J. Mao, *Angew. Chem., Int. Ed.* **2022**, 61, e202210789.

[5] M. Xia, X. Gong, W. Teng, H. Xiao, H. Li, H. Ou, G. Yang, *Appl. Catal. B* **2026**, 380, 125756.

[6] J. Yuan, W. Feng, Y. Zhang, J. Xiao, X. Zhang, Y. Wu, W. Ni, H. Huang, W. Dai, *Adv. Mater.* **2024**, 36, 2303845

[7] D. Hao, J. Ren, Y. Wang, H. Arandiyan, M. Garbrecht, X. Bai, H. K. Shon, W. Wei, B.-J. Ni, *Energy Mater. Adv.* **2021**, 2021, 9761236.

[8] D. Hao, Y. Wei, L. Mao, X. Bai, Y. Liu, B. Xu, W. Wei, B.-J. Ni, *J. Clean. Prod.* **2021**, 331, 129975.

[9] Z. Liu, S. Fan, X. Li, Z. Niu, J. Wang, C. Bai, J. Duan, M. O. Tadé, S. Liu, *Appl. Catal. B* **2023**, 372, 122416

[10] H.-T. Ren, S.-Y. Jia, J.-J. Zou, S.-H. Wu, X. Han, *Appl. Catal. B* **2015**, 176-177, 53.

[11] Z. Lian, D. Luo, J. Yang, Y. Yang, S. Tang, H. Li, D. Zhang, H. Li, *Angew. Chem., Int. Ed.* **2025**, e202516964.

[12] J. A. Anderson, *Catal. Today* **2012**, 181, 171.

[13] A. P. Varghese, R. T. P, B. Neppolian, A. S. AlArifi, S. K. Lakhera, *ACS Appl. Energy Mater.* **2024**, 7, 6527.

[14] Y. Zhao, J. Shen, J. Yuan, H. Mao, X. Cheng, Z. Xu, Z. Bian, *Nano Energy* **2024**, 124, 109499.

[15] X. Yan, Y. Wu, F. Guo, J. Xiong, Y. Zhang, L. Wang, J. Dai, G. Hao, W. Jiang, J. Di, *Adv. Funct. Mater.***2025**, 35, 2421669.

[16] Y. Xi, Y. Xiang, T. Bao, Z. Li, C. Zhang, L. Yuan, J. Li, Y. Bi, C. Yu, C. Liu, *Angew. Chem., Int. Ed.* **2024**, 63, e202409163.
